# Analgesic Effects of Triterpenoid Saponins From *Stauntonia chinensis* via Selective Increase in Inhibitory Synaptic Response in Mouse Cortical Neurons

**DOI:** 10.3389/fphar.2018.01302

**Published:** 2018-11-12

**Authors:** Su Chen, Yi Rong, Mengxue Liu, Song Cheng, Xiangming Liu, Xiaohong Li, Yi Yu, Guangzhong Yang, Xiaofei Yang

**Affiliations:** ^1^Key Laboratory of Cognitive Science, Hubei Key Laboratory of Medical Information Analysis and Tumor Diagnosis & Treatment, Laboratory of Membrane Ion Channels and Medicine, College of Biomedical Engineering, South-Central University for Nationalities, Wuhan, China; ^2^Gongqing Institute of Science and Technology, Gongqing, China; ^3^Department of Cancer, Liyuan Hospital, Tongji Medical College, Huazhong University of Science and Technology, Wuhan, China; ^4^Laboratory for Natural Products Chemistry, School of Pharmaceutical Sciences, South Central University for Nationalities, Wuhan, China

**Keywords:** anti-nociceptive activity, TSS, spontaneous release, hot-plate test, formalin test, capsaicin test

## Abstract

Triterpenoid saponins from *Stauntonia chinensis* (TSS) are potential therapeutic agents because of its analgesic properties. However, the underlying mechanisms of the anti-nociceptive activity of TSS are largely unclear, especially in CNS. The present study confirmed the analgesic effect of TSS using four models of acute pain based on thermal or chemical stimuli. TSS treatment specifically impaired the threshold of thermal- and chemical-stimulated acute pain. Naloxone did not block the anti-nociceptive effects of TSS, which showed no participation of the opioid system. We investigated the electrical signal in cultured cortical neurons to explore whether TSS treatment directly affected synaptic transmission. TSS treatment selectively increased spontaneous inhibitory synaptic release and GABA induced charge transfer in mouse cortical neurons. The effects of TSS were maintained for at least 8 h in cultured neurons and in injected mice. Taken together, our results suggest that the analgesic role of TSS in cortex occurs via a particular increase in the inhibitory synaptic response at resting state, which supports TSS as a potential candidate for inflammatory pain relief.

## Introduction

Pain reduces the quality of life, and it is an economic burden to society. Medically opioids are primarily used for pain relief, including anesthesia. But most opioid drugs are controlled substances because of their reputation for addiction and fatal overdose (Al-Hasani and Bruchas, 2011). NSAIDs, such as aspirin, naproxen, ibuprofen, and acetaminophen, are the most commonly used as analgesic drugs (Simon, 2013; Moore et al., 2014). However, gastrointestinal toxicity is reported as side-effect of NSAIDs for a long time (Bjarnason and Macpherson, 1989; Klasser and Epstein, 2005; Gargallo and Lanas, 2013). To explore safer candidates for analgesia, TCM is widely considered as an alternative medical system (Xue et al., 2010; Liu et al., 2015).

*Stauntonia chinensis* DC. (Lardizabalaceae), an evergreen herb growing in southern China, is commonly known as “Ye Mu Gua.” This herb is used as a TCM, especially for its anti-inflammatory and analgesic effects (Jiangsu New Medical College, 1977; Peng et al., 2003). The preparations, such as tablets and injections of *Stauntonia chinensis* DC, have been widely applied for the treatments of rheumatism arthralgia, postoperative analgesia, postherpetic and sciatic neuralgia clinically. Up to date, there have been several investigations aimed at studying the pharmacological mechanism of the anti-nociceptive and anti-inflammatory activities produced by *Stauntonia chinensis* and finding the specific material basis for these utilizations.

Ying et al. (2014) demonstrated that the 60% EtOH extract of *Stauntonia chinensis* was its active ingredients producing anti-nociceptive and anti-inflammatory activities. And the anti-inflammatory activity of *Stauntonia chinensis* extracts was related to the reduction of PGE2 production. However, the anti-nociceptive and anti-inflammatory activities of one fraction were not entirely consistent, which indicated that inhibition of the inflammatory response may be one of the anti-nociceptive mechanisms of *Stauntonia chinensis* extracts (Ying et al., 2014).

Similar results were observed for NSAIDs. The well-known mechanisms of NSAIDs in inflammatory pain therapy are the inhibition of COX-1 and COX-2, which decreases the production of prostanoids (Alhouayek and Muccioli, 2014; Díaz-González and Sánchez-Madrid, 2015; Dwivedi et al., 2015). Furthermore, the roles of NSAIDs in neurodegenerative disease have recently gained further attention (Ferencik et al., 2001; Iwata et al., 2004; Ajmone-Cat et al., 2008), which indicates that the drugs used to attenuate pain may have direct or indirect effects on brain neurons.

Ye et al. (2003) demonstrated that the *Stauntoniae* saponin exhibited affinity for myelin sheaths and axon membranes and resulted in nerve structure destruction and conduction blockage. The glycoside extracts from *Stauntonia chinensis* which contained triterpenoid saponins (percentage content was 52.4%) could protect injured spinal neurons and promote the growth of spinal neurons (Zhou and Ye, 2011). However, the mechanisms underlying the analgesic effects of triterpenoid saponins isolated from *Stauntonia chinensis* DC. (TSS) remain poorly understood, especially in central nerve system (CNS) (Jiangsu New Medical College, 1977).

The present study confirmed the analgesic effect of TSS using four models of acute pain based on thermal or chemical stimuli to clarify the direct effects of TSS treatment in the CNS.TSS treatment selectively increased spontaneous inhibitory synaptic transmission in cultured cortical neurons. Further investigation demonstrated a presynaptic, rather than postsynaptic, effect of TSS in neurons. The increased activity in inhibitory synapses lasted for at least 8 h after the TSS exposure. Moreover, except for acetic acid-induced writhing test, the hot-plate, formalin and capsaicin tests confirmed the analgesic effect 8 h after TSS injection as well. Naloxone did not block the anti-nociceptive effect of TSS in the hot-plate test, which demonstrated that the analgesic effect of TSS did not involve opioid receptor. Taken together, our results suggested that TSS could directly increase the inhibitory synaptic response in mice cortex neurons to enhance the thresholds of inflammatory pain. These results supported TSS as a potential candidate for inflammatory pain relief.

## Materials and Methods

### Extraction and Isolation of TSS

The stems of *Stauntonia chinensis* were collected from NanNing, Guangxi Zhuang Autonomous Region, China and identified by associate chief pharmacist Jin-Wei Huang at Guangxi Institute of Minority Medicine. A voucher specimen (20090801) was deposited with the Herbarium of School of Pharmaceutical Sciences, South Central University for Nationalities. According to the previously reported method (Xu et al., 2018), the stems of *Stauntonia chinensis* (2.5 kg) were extracted with 60% EtOH three times and then successively partitioned with EtOAc and *n*-BuOH. The extract of *n*-BuOH (109 g) was purified by HP-20 micro resin column chromatograpy to obtain TSS and the chemical composition of TSS was further analyzed by HPLC-ESI-MS/MS.

### Animals and Drugs

Male and female Kunming mice weighing 18–22 g were obtained from Hubei Research Center of Laboratory Animals [Grade SPF, SCXK (Hubei) 2015-0018] and acclimatized to the laboratory conditions for at least 1 week prior to experimentation. The mice were housed at a temperature of 22 ± 2°C under a 12 h light/12 h dark cycle (lights on at 7:00 AM, lights off at 7:00 PM) and maintained (5 animals per cage) with food and water *ad libitum*. Separate groups of mice were used for each analgesic test, and the animals were used only once in the experiments. All experimental procedures involving mice were performed under a protocol approved by the animal research ethics committee of South-Central University for Nationalities.

TSS was dissolved in isotonic (0.9% NaCl) saline and administered intraperitoneally at 2, 8, and 20 mg⋅kg^-1^, unless otherwise indicated. The following substances were used: morphine hydrochloride (Northeast Pharmaceutical Group Shenyang No. 1 Pharmaceutical Factory), naloxone hydrochloride (Beijing Hua Su Pharmaceutical Co., Ltd.), acetic acid, formalin, capsaicin (Sigma), and aspirin (Shenwei Pharmaceutical Co., Ltd.). All drugs were dissolved in isotonic saline, except capsaicin which was dissolved in absolute ethanol. The final concentration of ethanol did not exceed 10% and did not cause any *per se* effect. The dose volume of intraperitoneal injection was within a 10 ml⋅kg^-1^ level for mice.

### Hot Plate Test and Acetic Acid-Induced Writhing Test

The hot-plate test was used to measure response latencies according to the method described previously by Eddy and Leimbach (1953) with minor modification. Animals were placed into a glass cylinder and the time between placement and shaking or licking of the paws or jumping was recorded as the index of response latency. A full automatic thermal pain stimulator (BME-410C, Institute of Biomedical Engineering, Chinese Academy of Medical Sciences) was used. A cut-off time of 60 s was used to avoid tissue damage. Animals were selected 24 h in advance on the basis of reactivity. Mice that remained on the apparatus maintained at 55 ± 0.5°C less than 5 s and beyond 30 s were eliminated. The hot-plate test was performed four times at different time points post-drug administration. Each animal was tested prior to drug administration using the same heat stimulus to determine the baseline. Negative control animals received the isotonic saline used to dilute the drugs and morphine (5 mg⋅kg^-1^ intraperitoneally) was used as a positive control. Two groups of mice were pretreated with naloxone (1 mg⋅kg^-1^ intraperitoneally, a non-selective opioid receptor antagonist) 20 min prior to drug administration to assess the possible participation of the opioid system in the anti-nociceptive effect of TSS.

Abdominal constrictions were induced according to the procedures described previously by Spindola et al. (2010). Writhing movements were induced by an intraperitoneal injection of 0.8% acetic acid solution (10 ml⋅kg^-1^). Mice were individually placed into glass cylinders with a 20 cm diameter, and writhing movements were counted for 15 min after acetic acid injection. Animals were pretreated with different doses of TSS 30 min before the injection of acetic acid. Aspirin (20 mg⋅kg^-1^ intraperitoneally) was used as a positive control. Negative control animals received a similar volume of isotonic saline.

### Formalin and Capsaicin Tests

The procedure for formalin test was essentially the same as that described by Dubuisson and Dennis (1977). Twenty microliters of 5% formalin were injected into the dorsal surface of the right hind paw of mice. After the injection of formalin, the animals were immediately placed in a glass cylinder with a 20 cm diameter, and the time spent in licking the injected paw was monitored and recorded over for 0–5 min (early phase of licking) and 15–40 min (late phase of licking). The animals received different doses of TSS, aspirin (20 mg⋅kg^-1^ intraperitoneally) and isotonic saline 30 min prior to formalin injection.

The method for the capsaicin test followed the procedure of Corrêa et al. (1996). Capsaicin (20 μl) was injected intraplantarly (1.6 μg per paw) into the ventral surface of the right hind paw of mice. Animals were observed individually for 5 min following capsaicin injection. The amount of time spent licking the injected paw was recorded using a chronometer and was considered as indicative of nociception. The mice were given different doses of TSS 30 min prior to capsaicin injection. Control animals received aspirin (20 mg⋅kg^-1^ intraperitoneally) and isotonic saline.

### Cell Culture

The dissociated cortical neurons were prepared from randomly chosen P0 pups, as described previously (Gong et al., 2016). Briefly, the dissociated cortical neurons were dissected from P0 pups of WT Kunming mice, dissociated using 0.25% trypsin-EDTA digestion for 12 min at 37°C, plated on poly-L-lysine (Sigma)-coated 12-mm diameter circular glass coverslips, and cultured in MEM (Gibco) supplemented with 2 v⋅v^-1^% B27 (Gibco), 0.5 w⋅v^-1^% glucose, 100 mg/l transferrin, 5 v⋅v^-1^% fetal bovine serum (Gibco), and 2 mM Ara-C (Sigma). All animal procedures were performed in accordance with South-Central University for Nationalities animal use rules and the requisite approvals of animal use committees.

### mRNA Measurements and Immunocytochemistry

mRNA measurements were performed with RNA isolated from cultured neurons at 14–15 days *in vitro* (DIV14-15). RT-PCR reactions were performed in triplicate with GAPDH as an internal control.

Mouse cortical neurons were fixed in 4% paraformaldehyde and permeabilized with 0.2% Triton X-100, stained with anti-GAD65 (polyclonal; Sigma) and anti-MAP2 (monoclonal; Sigma) primary antibodies in PBS with 5% BSA, and visualized using Alexa Fluor 488 goat anti-rabbit and Alexa Fluor 546 goat anti-mouse secondary antibodies (Molecular Probes). Images were acquired using a Nikon C2 confocal microscope equipped with a 60× oil-immersion objective. Identical settings were used for all samples in each experiment. We measured the average pixel intensities via manually tracing each dendrite, with a >2-fold background signal.

### Electrophysiological Recordings

Electrophysiological recordings were performed in whole-cell patch-clamp mode using concentric extracellular stimulation electrodes. Patch pipettes were pulled from borosilicate glass capillary tubes (World Precision Instruments, Inc.) using a P-97 pipette puller. The resistance of pipettes filled with intracellular solution varied between 3 and 5 mOhm. After formation of the whole-cell configuration and equilibration of the intracellular pipette solution, the series resistance was adjusted to 8–10 mOhm. The whole-cell pipette solution contained 120 mmol⋅l^-1^ CsCl, 10 mmol⋅l^-1^ HEPES, 10 mmol⋅l^-1^ EGTA, 0.3 mmol⋅l^-1^ Na-GTP, 3 mmol⋅l^-1^ Mg-ATP and 5 mmol⋅l^-1^ QX-314 (pH 7.2, adjusted with CsOH). The bath solution contained 140 mmol⋅l^-1^ NaCl, 5 mmol⋅l^-1^ KCl, 2 mmol⋅l^-1^ MgCl_2_, 2 mmol⋅l^-1^ CaCl_2_, 10 mmol⋅l^-1^ HEPES-NaOH, and 10 mmol⋅l^-1^ glucose (pH 7.4).

In all the recordings, neurons were voltage-clamped at -70 mV. Synaptic currents were monitored with an EPC10 amplifier (HEKA). Evoked synaptic responses were recorded using a bipolar electrode placed 100–150 mm from the soma of neurons. Single extracellular stimulus pulses (90 μA, 1 ms) were controlled with a Model 2100 Isolated Pulse Stimulator (A-M Systems, Inc.) for all evoked measurements. IPSCs and EPSCs were pharmacologically isolated with the addition of the AMPA and NMDA receptor blockers CNQX (20 μmol⋅l^-1^) and AP-5 (50 μmol⋅l^-1^), respectively, or the GABAA receptor blocker picrotoxin (50 μmol⋅l^-1^) in the extracellular solution. Spontaneous mIPSCs or mEPSCs were monitored in the presence of tetrodotoxin (TTX, 1 μmol⋅l^-1^) to block action potentials. The data were digitized at 10 kHz with a 2-kHz low-pass filter. Miniature events were analyzed in Clampfit 10 (Molecular Devices) using the template-matching search and a minimal threshold of 5 pA. Each event was visually inspected for inclusion or rejection by an experimenter who was blinded to the recording condition. Sucrose-evoked release was triggered via a 30-s application of bath solution to which 0.5 mol⋅l^-1^ sucrose was added and also contained AP-5, CNQX, and TTX. GABA-evoked release was measured via a 20-s application of bath solution to which 200 μmol⋅l^-1^ GABA was added. McN-A-343 (4-3-chlorophenyl-carbamoyloxy-2-butynyltrimethylammonium, 50 μmol⋅l^-1^) was dissolved in bath solution just before experiment.

### Statistical Analysis

The data were expressed as the mean ± SEM and analyzed with SPSS software (version 17.0) and Prism 6.01 (GraphPad). All results were submitted to one-way analysis of variance (ANOVA), considering as critical level *P* < 0.05 to evaluate significant difference between the control and treated groups, followed by Dunnett’s *t*-test (for Figures 2, 6) or to Student’s *t*-test comparing each condition to the indicated control experiment. All of which are described in figure legends.

## Results

### Identification of Triterpenoid Saponins in the TSS by HPLC-ESI-MS/MS

**FIGURE 1:**
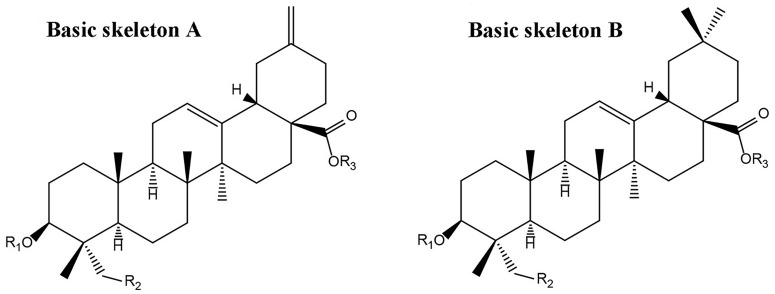
Basis skeletons of chemical structures of triterpene saponins identified in TSS.

According to the previously reported results (Xu et al., 2018), nearly 36 peaks were detected from TSS. Twenty of these major peaks were identified by comparison of the molecular formulae and fragmentation patterns with reported data in the literature. Their structures of triterpene saponins were determined as depicted in Figure 1 and Table 1.

**Table 1:** Chemical structures of triterpene saponins identified in TSS.

Compound	Basic skeleton	R_1_	R_2_	R_3_
1	A	-Ara(2←1)GluA	OH	-Glc(6←1)Glc(4←1)Rha
2	A	-Ara[(2←1)Rha] (3←1)Ara	OH	-Glc(6←1)Glc(4←1)Rha
3	A	-Ara(2←1)Rha	OH	-Glc(6←1)Glc(4←1)Rha
4	A	-Ara	OH	-Glc(6←1)Glc(4←1)Rha
5^∗^	A	-Ara(2←1)Rha	OH	-Glc(6←1)Glc
6	B	-Ara(2←1)GluA	OH	-Glc(6←1)Glc(4←1)Rha
7	B	-Ara[(2←1)Rha] (3←1)Ara	OH	-Glc(6←1)Glc(4←1)Rha
8	B	-Ara(2←1)Rha	OH	-Glc(6←1)Glc(4←1)Rha
9	B	-Ara	OH	-Glc(6←1)Glc(4←1)Rha
10	B	-Ara[(2←1)Rha] (3←1)Ara	OH	-Glc(6←1)Glc
11	B	-Ara(2←1)Rha	OH	-Glc(6←1)Glc
12	A	-Ara[(2←1)Rha] (3←1)Xyl	H	-Glc(6←1)Glc(4←1)Rha
13	A	-Ara(2←1)Rha	H	-Glc(6←1)Glc(4←1)Rha
14	A	-Ara[(2←1)Rha] (3←1)Ara	H	-Glc(6←1)Glc
15	A	-Ara(2←1)Rha(3←1)Xyl	OH	H
16^∗^	B	-Ara[(2←1)Rha] (3←1)Ara(3←1)Ara	OH	H
17^∗^	B	-Ara[(2←1)Rha] (3←1) GluA	OH	H
18	B	-Ara(2←1)Rha	OH	H
19	A	-Ara[(2←1)Rha] (3←1)Ara	H	H
20	B	-Ara[(2←1)Rha] (3←1) GluA	H	H

*^∗^New compound*.

The aglycones were mainly composed of hederagenin, 30-norhederagenin, akebonic acid and oleanic acid, and the sugar chain is composed of glucose, rhamnose, arabinose, xylose and glucuronic acid. The chromatographic behaviors of TSS in reverse phase chromatography depended on the polarities of their substituents. In general, bidesmoside triterpenoid glycosides (compounds **1**–**14**) exhibited longer retention times than monodesmosides (compounds **15**–**20**). On the other hand, TSS containing a 30-norhederagenin aglycon (compounds **1**–**5**) eluted more rapidly than those containing a hederagenin aglycon (compounds **6**–**11**). If TSS contain the same aglycon and sugar chain ether-linked to C-28(e.g., compounds **2**–**4** and **7–9**), then the retention time became shorter with the increase in the number of sugars attached at C-3. However, compounds **1** and **6**, which contained glucuronic acid, were exceptions due to their higher hydrophilicities.

### TSS Increased the Reaction Time of Mice in Hot Plate Test

To observe the nociceptive activity of TSS on thermal-stimulated acute pain, five groups of 10 mice were respectively treated with isotonic saline, morphine and three doses of TSS. TSS (2 and 8 mg⋅kg^-1^) did not produce significant alteration in the reaction time 30, 60, and 90 min after the administration. TSS (20 mg⋅kg^-1^), however, produced a significant increase in the reaction time 30 min after the administration. Morphine, used as reference drug, produced a significant and marked analgesic effect in both models. The other two groups of 10 mice were pretreated with naloxone followed by morphine or 20 mg⋅kg^-1^ TSS. Naloxone largely reversed the anti-nociception caused by injection of morphine, but it did not significantly alter the anti-nociceptive action of TSS. The detailed results were shown in Table 2. Our results showed that TSS could increase the threshold of thermal-stimulated acute pain via a mechanism that was not related to opioid receptor.

**Table 2:** Effect of TSS on hot plate test in mice.

Group	Dose(mg/kg)	Reaction time(s)			
		Before administration	30 min after administration	60 min after administration	90 min after administration
isotonic saline	/	15.8 ± 4.5	15.7 ± 4.5	16.8 ± 5.6	17.5 ± 5.2
Morphine	5	14.8 ± 2.0	27.3 ± 3.8^∗^	27.0 ± 4.9^∗^	24.7 ± 4.2^∗^
TSS	2	13.9 ± 5.5	14.8 ± 3.9	17.1 ± 4.1	15.1 ± 3.1
TSS	8	15.8 ± 4.9	16.5 ± 5.4	18.0 ± 5.8	18.7 ± 5.4
TSS	20	15.9 ± 2.1	24.6 ± 3.4^∗^	16.8 ± 4.3	18.1 ± 4.5
Morphine and naloxone	5 (morphine) 1(naloxone)	14.8 ± 1.7	16.0 ± 4.9	16.7 ± 5.2	15.9 ± 3.2
TSS and naloxone	20(TSS) 1(naloxone)	16.9 ± 2.2	24.9 ± 4.2^∗^	19.0 ± 5.3	17.1 ± 5.1

*^∗^*P* < 0.05 versus isotonic saline group (*n* = 10)*.

### TSS Reduced Acetic Acid-Induced Writhing Movements of Mice

To discover the nociceptive activity of TSS on chemical-stimulated acute visceral pain, five groups of 10 mice were respectively treated with isotonic saline, aspirin and three doses of TSS. In the acetic acid-induced writhing test, compared with isotonic saline treatment, TSS (8 and 20 mg⋅kg^-1^) and aspirin decreased the number of acetic acid-induced writhing movements (Table 3), which indicated that TSS could relieve acetic acid-induced acute visceral pain.

**Table 3:** Effect of TSS on acetic acid-writhing in mice.

Group	Dose(mg/kg)	Mean number of writhing mouse 15 min
Isotonic saline	/	40.3 ± 5.4
Aspirin	20	10.6 ± 2.6^∗^
TSS	2	37.0 ± 3.7
TSS	8	26.5 ± 5.6^∗^
TSS	20	24.0 ± 4.2^∗^

*^∗^*P* < 0.05 versus isotonic saline group (*n* = 10)*.

### TSS Decreased the Time Spent in Licking the Injected Paw of Mice on Formalin and Capsaicin Tests

**FIGURE 2:**
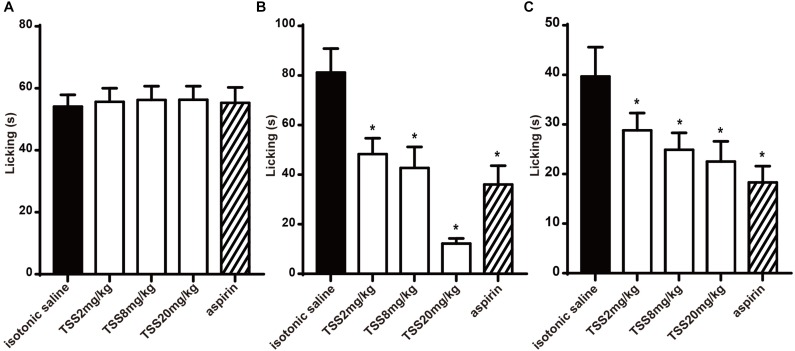
Effect of TSS on formalin and capsaicin test. **(A)** Effect of TSS against formalin-induced licking in the first phase in mice. **(B)** Effect of TSS against formalin-induced licking in the second phase in mice. **(C)** Effect of TSS against capsaicin-induced licking in mice. Each column represented the mean of the values obtained in 10 mice and the error bars indicate the SEM. The closed columns indicated the control value (isotonic saline group), the open columns correspond to mice treated with TSS and the twill column indicated the positive control value (aspirin group). ^∗^Denote the significance levels, when compared with control group (one-way ANOVA), *P* < 0.05.

For understanding the nociceptive activity of TSS on chemical-stimulated acute pain, five groups of 10 mice were respectively treated with isotonic saline, aspirin and three doses of TSS. In the formalin test, the time spent in licking the injected paw in the first and second phases were 54.1 ± 3.8 and 81.2 ± 9.7 s respectively in control mice. Figure 2A showed that TSS and aspirin did not reduce the time spent in licking the injected paw in the first phase. However, TSS caused a marked and dose-related inhibition in the second phase of formalin-induced licking (Figure 2B). Aspirin was also anti-nociceptive in the second phase. Results showed that TSS could relieve formalin -induced acute inflammatory pain.

In the capsaicin test, five groups of 10 mice were respectively treated with isotonic saline, aspirin and three doses of TSS. TSS decreased the time spent in licking the injected paw in a dose-dependent manner. The capsaicin-induced pain was also inhibited significantly by aspirin (Figure 2C). These results showed that TSS could relieve capsaicin-induced acute neuropathic pain.

### TSS Exposure Selectively Increased Spontaneous Inhibitory Synaptic Response

**FIGURE 3:**
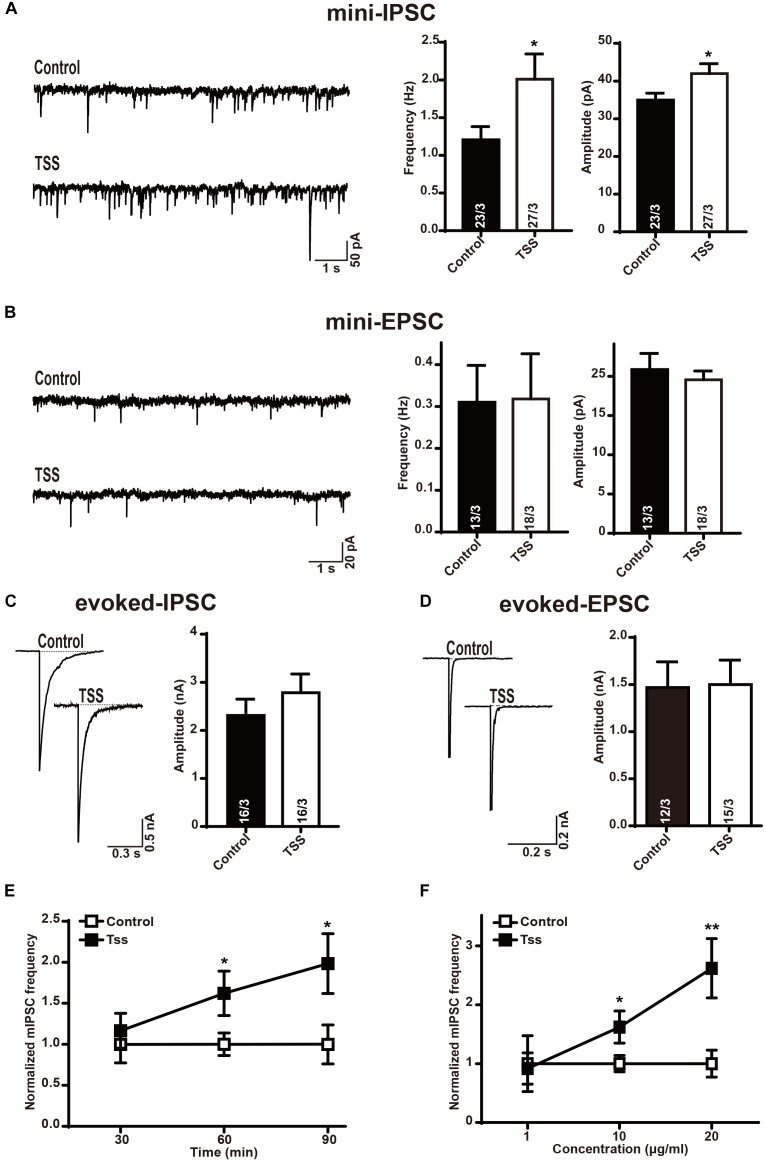
TSS treatment selectively enhanced the response of inhibitory synapses. **(A)** Sample traces (left) and summary graphs of the frequency (middle) and amplitude (right) of mIPSCs recorded in cultured cortical neurons treated with (TSS) or without (Control) 10 μg⋅ml^-1^ TSS for 60 min. **(B)** Sample traces (left) and summary graphs of the frequency (middle) and amplitude (right) of mEPSCs recorded in neurons as described for **(A)**. **(C)** Sample traces (left) and summary graphs of the amplitude (right) of eIPSCs recorded in neurons as described for **(A)**. **(D)** Sample traces (left) and summary graphs of the amplitude (right) of eEPSCs recorded in neurons as described for **(A)**. **(E)** Plot of cultured cortical neurons treated with 10 μg⋅ml^-1^ TSS for 30, 60, and 90 min, respectively. **(F)** Plot of cortical neurons treated with 1 μg⋅ml^-1^, 10 μg⋅ml^-1^ and 20 μg⋅ml^-1^ TSS for 60 min, respectively. Data shown in summary graphs are means ± SEM. For **(A–D)**, numbers of cells/independent cultures analyzed are listed in the bars. Three independent experiments were performed for E and F. Statistical assessments were performed by the Student’s *t*-test comparing each condition to the indicated control experiment (^∗^*P* < 0.05, ^∗∗^*P* < 0.01).

Considering the TSS treatment might affect the brain directly in the process of pain relief, we measured the neurotransmitter release in cultured mouse cortical neurons. To confirm our cultured neurons were capable to exhibit the synaptic activity properly and sensitive to certain drugs that altered synaptic transmission, McN-A-343 (Koga et al., 2017), which is selective for the muscarinic M_1_ receptor, was used. Similarly, the frequency but not amplitude of mIPSCs in our cultured neurons was increased by McN-A-343 (Supplementary Figure S1), which indicated that our cultured neurons were able to precisely reflect synaptic release and alternation. After confirming the availability of our cortical neurons, the spontaneous response was characterized after incubation with 10 μg⋅ml^-1^ TSS for 60 min. Interestingly, the frequency and amplitude of mIPSCs were both significantly increased by TSS (Figure 3A). Whereas the frequency and amplitude of mEPSCs were not altered (Figure 3B), which suggested that TSS could act on brain neurons directly. To further test the effects of TSS on primary neurons, the action-potential evoked-IPSCs and evoked-EPSCs mediated by AMPARs were measured. Similarly, the amplitude of evoked-EPSCs was unchanged by 10 μg⋅ml^-1^ TSS treatment (Figure 3D), which confirmed that TSS had no effect on excitatory terminals. On the other hand, the amplitude of evoked-IPSCs was slightly, although not significantly, increased by 10 μg⋅ml^-1^ TSS (Figure 3C). This result confirmed that TSS affected the inhibitory synaptic response. However, the difference between spontaneous and action-potential evoked responses in inhibitory synapses suggested that TSS played a preferential role at the resting stage to continuously increase the threshold of pain at basal levels rather than transiently altering the threshold of pain with stimuli.

Then we asked if the effects of TSS in inhibitory synapses were time or dose dependent. For this purpose, the frequency of mIPSCs was tested after different time of TSS exposure. Our results revealed that 60 or 90 min of 10 μg⋅ml^-1^ TSS exposure increased the frequency of mIPSCs but 30 min of TSS treatment did not (Figure 3E), indicating the increasing threshold of pain reflected by an increase inhibitory synaptic response induced by TSS took 1 h or so. On the other hand, the frequency of mEPSCs was kept unchanged with 30, 60, or 90 min of 10 μg⋅ml^-1^ TSS treatment (Supplementary Figure S2), confirming the effects of TSS were specific in inhibitory synapses. Therefore, 60 min TSS exposure was used for all the following experiments. Moreover, the effects of different concentration of TSS were next quantitated. 10 μg⋅ml^-1^ or 20 μg⋅ml^-1^ but not 1 μg⋅ml^-1^ TSS treatment increased the frequency of mIPSCs (Figure 3F), suggesting a dose dependent regulation of TSS treatment. To minimize any potential side-effect of TSS treatment, we used 10 μg⋅ml^-1^ TSS, the concentration could already increase the threshold of pain, for all the following measurements.

### TSS Treatment Increased the Inhibitory Presynaptic Release Probability

**FIGURE 4:**
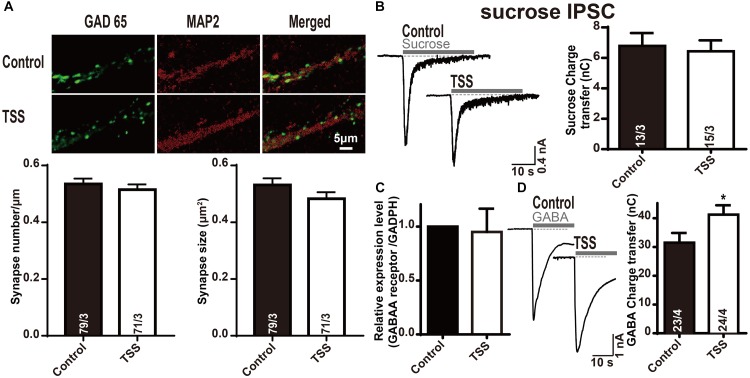
The postsynaptic GABA response was specifically increased by TSS treatment. **(A)** Representative images (up) and summary graphs of synapse density (left lower) and cluster size (right lower) in cultured mouse cortical neurons treated with (TSS) or without (Control) 10 μg⋅ml^-1^ TSS for 60 min. The scale bar in right lower corner applies to all images. **(B)** Sample traces (left) and summary graphs (right) of IPSCs evoked by 0.5 mol⋅l^-1^ sucrose, recorded in neurons as described for **(A)**. **(C)** Measurements of GABAA receptor mRNA levels in neurons as described for **(A)**. **(D)** Sample traces (left) and charge transfer (right) induced by application of 200 μmol⋅l^-1^ GABA in neurons as described for **(A)**. Data shown in summary graphs are means ± SEM. For **(AB,D)**, numbers of cells/independent cultures analyzed are listed in the bars. Four independent experiments were performed for **(C)**. Statistical assessments were performed by the Student’s *t*-test comparing each condition to the indicated control experiment (^∗^*P* < 0.05).

The increase in the frequency and amplitude of mIPSCs induced by TSS treatment indicated both pre- and postsynaptic roles for TSS. Therefore, the pre- and postsynaptic effects of TSS were differentiated. We stained neurons with antibodies to MAP2 and glutamic acid decarboxylase 65 (GAD65, an inhibitory presynaptic marker) to selectively visualize inhibitory nerve terminals. Quantitation of immunofluorescence revealed that the density and size of inhibitory synapses were unchanged after TSS treatment (Figure 4A), which suggested that the synapses themselves were not altered by TSS. The size of the RRP of vesicles from the synaptic responses induced by application of a hypertonic sucrose solution (Rosenmund and Stevens, 1996) was further tested. Similarly, no obvious difference in RRP size was detected with TSS treatment (Figure 4B), which indicated that the presynaptic priming ability was not impaired by TSS. Since the synapses number and the amount of releasable vesicles at each synapse were not altered by TSS, the most possible reason for increased mIPSCs frequency was an increase in the release probability after TSS treatment. Moreover, the slight increase observed in eIPSCs amplitude also consistent with this hypothesis.

### TSS Treatment Enhanced GABA Receptor Activity

After demonstrating the presynaptic effect of TSS, we investigated whether TSS had postsynaptic effect as well. We performed quantitative RT-PCR measurements of GABAA receptor in cultured cortical neurons with or without TSS exposure. The measured relative mRNA levels were normalized to GAPDH (Figure 4C). Our results revealed that TSS treatment did not change the expression of GABAA receptor. To test whether TSS treatment directly affected postsynaptic GABAergic response, we then measured GABA-induced charge transfer. Interestingly, TSS exposure significantly increased the total charge transfer with 200 μmol⋅l^-1^ GABA application (Figure 4D). Therefore, our results clarified that TSS treatment increased GABA induced charge transfer but not the expression of GABAA receptor, which indicated an increased postsynaptic GABAergic response induced by TSS.

### Effects of TSS at Inhibitory Synapses Sustained at Least 8 h

**FIGURE 5:**
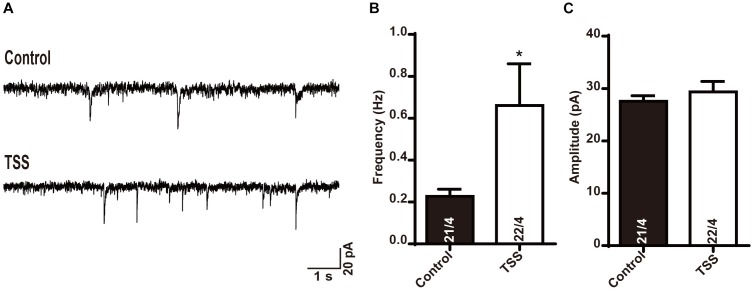
The effect of TSS on inhibitory synaptic transmission was maintained for 8 h. Sample traces **(A)** and summary graphs of the frequency **(B)** and amplitude **(C)** of mIPSCs recorded in cultured mouse cortical neurons with 8 h recovery after treated with (TSS) or without (Control) 10 μg⋅ml^-1^ TSS for 60 min. Data shown in summary graphs are means ± SEM. Numbers of cells/independent cultures analyzed are listed in the bars. Statistical assessments were performed by the Student’s *t*-test comparing each condition to the indicated control experiment (^∗^*P* < 0.05).

Since TSS treatment increased the basal inhibitory response in brain neurons to enhance the threshold of pain, we investigated the effective time period of TSS. We first treated cultured neurons with 10 μg⋅ml^-1^ TSS for 60 min, and then incubated the neurons in normal growth medium for different time. Our results revealed that the increased frequency of mIPSCs induced by TSS treatment was observed after 4 (Supplementary Figure S3A) and 8 h (Figures 5A–C) of recovery. However, the increase in mIPSCs frequency vanished after 12 h of recovery (Supplementary Figure S3B). Therefore, our results suggested that the increased threshold induced by TSS sustained for at least 8 h. Interestingly, not like the mIPSCs frequency, the amplitude of mIPSCs was not increased after recovery (Figures 5A–C and Supplementary Figure S3). Therefore, the presynaptic effect reflected as increased mIPSCs frequency kept longer than the postsynaptic effect reflected as increased mIPSCs amplitude, which suggested that the presynaptic effect was the dominant role of TSS.

### Effects of TSS in Hot Plate, Formalin and Capsaicin Tests Sustained for 8 h

**FIGURE 6:**
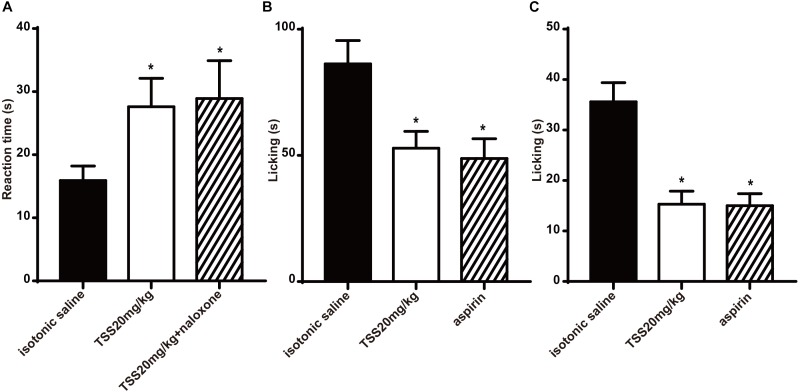
Effects of TSS in hot plate, formalin and capsaicin test sustained at least 8 h. **(A)** Effect of TSS on thermal-stimulated acute pain 8 h after the administration and the pretreatment of mice with naloxone did not change the anti-nociceptive action caused by TSS. **(B)** Effect of TSS against formalin-induced licking in the second phase in mice 8 h after the administration. **(C)** Effect of TSS against capsaicin-induced licking in mice 8 h after the administration. Each column represented the mean of the values obtained in 10 mice and the error bars indicate the SEM. The closed columns indicated the control value (isotonic saline group), the open column correspond to mice treated with TSS and the twill column indicated the positive control value. ^∗^Denote the significance levels, when compared with control group (one-way ANOVA), *P* < 0.05.

To further confirm the effective time of TSS, we investigated whether TSS (20 mg⋅kg^-1^ intraperitoneally) exhibited an anti-nociceptive effect 8 h after the administration using hot plate test, acetic acid-induced writhing test, formalin test and capsaicin test. In hot plate test, TSS produced a significant increase in the reaction time 8 h after the administration and the pretreatment of mice with naloxone did not change the anti-nociceptive action of TSS (Figure 6A). Our previous results showed that TSS (20 mg⋅kg^-1^) could only induce an increase in the reaction time 30 min but not 60 or 90 min after the administration in hot plate test. The short term and long term effects of TSS seem contradictory. To figure out the nature of TSS treatment, we re-selected 30 mice and tested their reaction times 30, 60, 90 min, 2, 4, and 8 h after the TSS (20 mg⋅kg^-1^) administration in parallel, respectively. Consistently, an increased reaction time was observed 30 min but not 60 or 90 min after the administration. However, the analgesic effect re-appeared after 2 h and remained until 8 h (Supplementary Figure S4). One possible explanation was that the analgesic effect of TSS in hot plate test had one fast phase and another slow but long lasting phase. These two phases of analgesic effects may occur via different modulation mechanisms, and cortex was likely involved in the slow phase of the 60-min or longer reaction time.

However, in acetic acid-induced writhing test, TSS could not change the number of acetic acid-induced writhing movements 8 h after the administration. The mean numbers of acetic acid-induced writhing movements 8 h after the administration in isotonic saline group and TSS group were 38.0 ± 6.5 and 42.9 ± 4.4, respectively (*P* > 0.05). In formalin test and capsaicin test, TSS reduced the time spent in licking the injected paw with formalin in the second phase (Figure 6B) and that induced by capsaicin (Figure 6C). All the results showed TSS could relieve thermal-stimulated pain, chemical-stimulated inflammatory pain and chemical-stimulated neuropathic pain except for chemical-stimulated visceral pain 8 h after the administration.

## Discussion

Nociceptive pain is an important early warning sign to avoid tissue damage caused by intense, potentially harmful stimuli. Electrical activity in nociceptive pain is initiated via ion channels such as the TRPV1 and voltage-gated sodium channels (Laedermann et al., 2013; Mickle et al., 2015). However, neuropathic and inflammatory pain occur after tissue damage (Marchand et al., 2005). Multiple chemical mediators are released from injured cells and recognized by receptors, which increases the sensitivity of neurons and aggravates the pain (Basbaum et al., 2009; Chen et al., 2013).

The sensitivity of primary nociceptors was enhanced by PKA and PKC pathway activated by the interaction of inflammatory mediators and GPCRs in the periphery. On the other hand, dorsal horn neurons increased the excitability and then transferred the signal to brain. Glutamate receptors, which include ionotropic AMPA, NMDA and kainate receptors and metabotropic mGluRs, may underline the increased excitability (Dougherty and Willis, 1992; Schaible et al., 2002). AMPA and NMDA receptor antagonists reduced the sensitivity of spinal neurons (Schaible et al., 1991; Dougherty et al., 1992; Ren et al., 1992). Ca^2+^ influxes also activated intracellular phosphorylation signal pathways to phosphorylate glutamate receptors, which modulated the activity or localization of these receptors (Caudle et al., 2005; Ji et al., 2009). Therefore, Ca^2+^ dependent synaptic release was clearly involved in the regulation of neuropathic and inflammatory pain. Needless to say, a downregulation of the hypersensitivity of peripheral terminals and brain neurons is a common method to relieve neuropathic and inflammatory pain.

In the pain therapy, TCM was considered as a promising alternate. The analgesic effects of TCM were intensively investigated due to their safety and efficacy. For instance, Wu-tou decoction, a classic TCM formula with anti-inflammatory activity, was explored to reduce the expression of TRPV1 and the TRPA1 in dorsal root ganglia, therefore inhibiting chronic pain (Wang et al., 2015). The aqueous extract of Flos populi exhibited significant anti-inflammatory activity against cotton pellet-induced granuloma and analgesic activity in the hot-plate test (Xu et al., 2014). Moreover, in the formalin assay, dehydrocorybulbine reduced the time spent licking in both early and late phase, which indicated a role in anti-nociceptive activity (Zhang et al., 2014). Thousands of years of use accumulated a large number of effective compounds of TCM, which offered an opportunity to identify new analgesic compounds.

Here, we demonstrated that 30-min TSS administration effectively increased the reaction time of mice in hot plate test, decreased the number of acetic acid-induced writhing movements of mice, and reduced the time spent licking the paw injected with formalin and capsaicin, which supported an analgesic activity of TSS. To further explore the analgesic mechanisms of TSS in brain, we recorded the neurotransmitter release after TSS treatment in mouse cortical neurons and found that the spontaneous inhibitory activity was selectively increased by TSS. The postsynaptic response induced by application of GABA was also increased by TSS. Moreover, we demonstrated that the analgesic activity of TSS could sustain for 8 h. The pretreatment of mice with naloxone did not change the anti-nociceptive action of TSS, which suggested that the opioid system did not take participation in the anti-nociceptive effect of TSS. Therefore, our results clarified that the analgesic effect of TSS in the brain was due to the enhanced activating threshold caused by increased inhibitory synaptic response, especially in the long term regulation.

Why is cortex important in the pain modulation? Pain, mediated by multiple cellular and molecular mechanisms, was extremely complicated. One single mechanism or area is hard to explain pain at all levels from spinal cord to brain. There were many regions in nerve system involved in pain, such as spinal cord, periaqueductal gray, amygdaloid nucleus and so on. However, the importance of cortex in pain modulation was contiguously reported. Different cortical regions such as ACC, insular cortex, primary and secondary somatosensory cortex and prefrontal cortex could be activated by various noxious stimuli (Talbot et al., 1991; Apkarian et al., 2005; Bushnell et al., 2013; Bliss et al., 2016). Altering the hyperexcitability of cortical pyramidal neurons played a role in the development of neuropathic pain (Xiong et al., 2017). Painful stimuli disturbed the balance between excitatory and inhibitory synaptic transmission in ACC (Gong et al., 2010). Here, we revealed that TSS treatment altered the synaptic activity of cultured mouse cortical neurons, which confirmed the involvement of cortex in neuropathic and inflammatory pain. However, our results did not exclude the possibility of the involvement of other regions in pain modulation.

The selective increase in spontaneous inhibitory synaptic responses induced by TSS raised another question that whether the E/I balance directly correlated to the regulation of pain sense. Glutamate and GABA systems were known to generate the opposite forces in neurotransmission and played key roles to the proper function of the CNS. Previous studies showed that α-asarone enhanced GABAergic neurotransmission but decreased glutamatergic release to restore the E/I balance and inhibit the chronic inflammatory pain induced by hind-paw injection of complete Freund’s adjuvant (Tian et al., 2017). The expression level of cytoplasmic polyadenylation element binding protein 1 was reported to correlate to presynaptic E/I release in the regulation of chronic pain (Yue et al., 2017). Furthermore, E/I balance was suggested to play a role in vestibular migraine (Espinosa-Sanchez and Lopez-Escamez, 2015). The present study revealed that both presynaptic inhibitory release probability and postsynaptic GABA receptor activity were enhanced by TSS treatment, whereas the glutamatergic neurotransmission was unchanged, which confirmed that the importance of E/I balance in the regulation of neuropathic and inflammatory pain.

## Conclusion

Our observations supported the use of TSS as an analgesic agent. Further investigations are needed to isolate specific effective analgesic components from TSS and determine the underlying molecular mechanisms of the components in analgesia.

## Ethics Statement

We indicate that the research were conducted under a protocol approved by the animal research ethics committee of South-Central University for Nationalities. All animal experiments were carried out in accordance with the National Institutes of Health guide for the care and use of Laboratory animals (NIH Publications No. 8023, revised 1978).

## Author Contributions

SuC and YR carried out the experiments and wrote the manuscript. ML, SoC, XiaoL, and YY performed the study. XianL, GY, and XY contributed to the planning of the work and wrote the paper.

## Conflict of Interest Statement

The authors declare that the research was conducted in the absence of any commercial or financial relationships that could be construed as a potential conflict of interest.

## Abbreviations

ACC: anterior cingulate cortex
AMPARs: AMPA-type glutamate receptors
E/I: excitation/inhibition
eIPSCs: evoked inhibitory postsynaptic currents
GAD65: glutamic acid decarboxylase 65
mEPSCs: miniature excitatory postsynaptic currents
mGluRs: G-protein-coupled glutamate receptors
mIPSCs: miniature inhibitory postsynaptic currents
NSAIDs: non-steroidal anti-inflammatory drugs
P0: postnatal day 0
RRP: readily releasable pool
TCM: traditional Chinese medicine
TRPA1: transient receptor potential A1
TRPV1: transient receptor potential cation channel subtype V1
TSS: triterpenoid saponins from *Stauntonia chinensis*
