# Supplementary material for: Analgesic Effects of Triterpenoid Saponins From Stauntonia chinensis via Selective Increase in Inhibitory Synaptic Response in Mouse Cortical Neurons
Source: Front Pharmacol. 2018 Nov 12;9:1302. doi: 10.3389/fphar.2018.01302 (PMC6241161; doi:10.3389/fphar.2018.01302)
Supplement: Supplementary file 1 [file Data_Sheet_1.pdf]

Supplementary Figure 1. McN-A-343 increased the frequency of mIPSCs in cultured cortical neurons.

Sample traces (A) and summary graphs of the normalized frequency (B, left) and amplitude (B, right) of mIPSCs recorded in cultured mouse cortical neurons treated with 50 ¦Ìmol·l^{-1} McN-A-343. Data shown in summary graphs are means ± SEM; numbers of cells/independent cultures analyzed are listed in the bars. Statistical assessments were performed by the Student's t test comparing each condition to the indicated control experiment (*P < 0.05).

Supplementary Figure 2. The frequency of mEPSCs was unaltered by TSS treatment for different time. (for Figure 3)

(A) Plot of cultured cortical neurons treated with 10 ¦Ìg·ml^{-1} TSS for 30, 60 and 90 mins, respectively. 3 independent experiments were performed. Statistical assessments were performed by the Student's t test comparing each condition to the indicated control experiment.

Supplementary Figure 3. The effect of increased inhibitory response by TSS treatment was retained after 4 hrs but not 12 hrs recovery. (for Figure 5)

(A) Sample traces (left) and summary graphs of the frequency (middle) and amplitude (right) of mIPSCs recorded in cultured mouse cortical neurons with 4 hrs recovery after treated with (TSS) or without (Control) 10 ¦Ìg·ml^{-1} TSS for 60 mins. (B) Sample traces (left) and summary graphs of the frequency (middle) and amplitude (right) of mIPSCs monitored in cultured mouse cortical neurons with 12 hrs recovery after treated with (TSS) or without (Control) 10 ¦Ìg·ml^{-1} TSS for 60 mins. Data shown in summary graphs are means ± SEM; numbers of cells/independent cultures analyzed are listed in the bars. Statistical assessments were performed by the Student's t test comparing each condition to the indicated control experiment (*P < 0.05).

Supplementary Figure 4. Effects of TSS at different time after the administration on hot plate test. (for Table 2 and Figure 6)

Each column represented the mean values obtained in 30 mice and the error bars indicated the S.E.M. * denote the significance levels, when compared with the reaction time before the administration (one-way ANOVA followed by Dunnett's *t*-test), $P<0.05$.

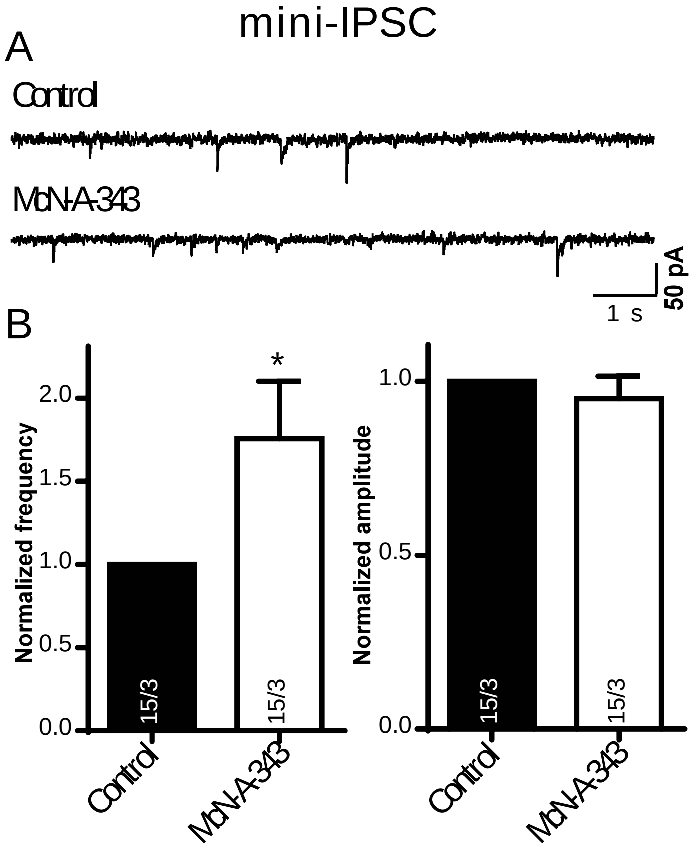

mini-IPSC
A
Control
McN-A-343
50 pA
1 s
B
Normalized frequency
2.0
1.5
1.0
0.5
0.0
*
15/3
15/3
Control
McN-A-343
Normalized amplitude
1.0
0.5
0.0
15/3
15/3
Control
McN-A-343

A

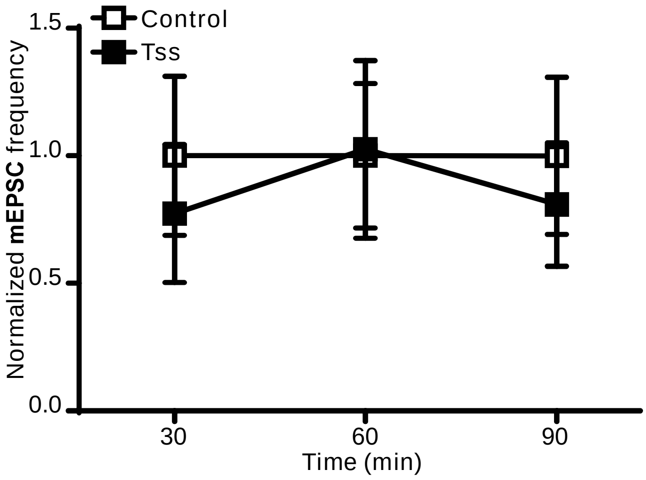

Control
Tss
1.5
1.0
0.5
0.0
Normalized mEPSC frequency
30
60
90
Time (min)

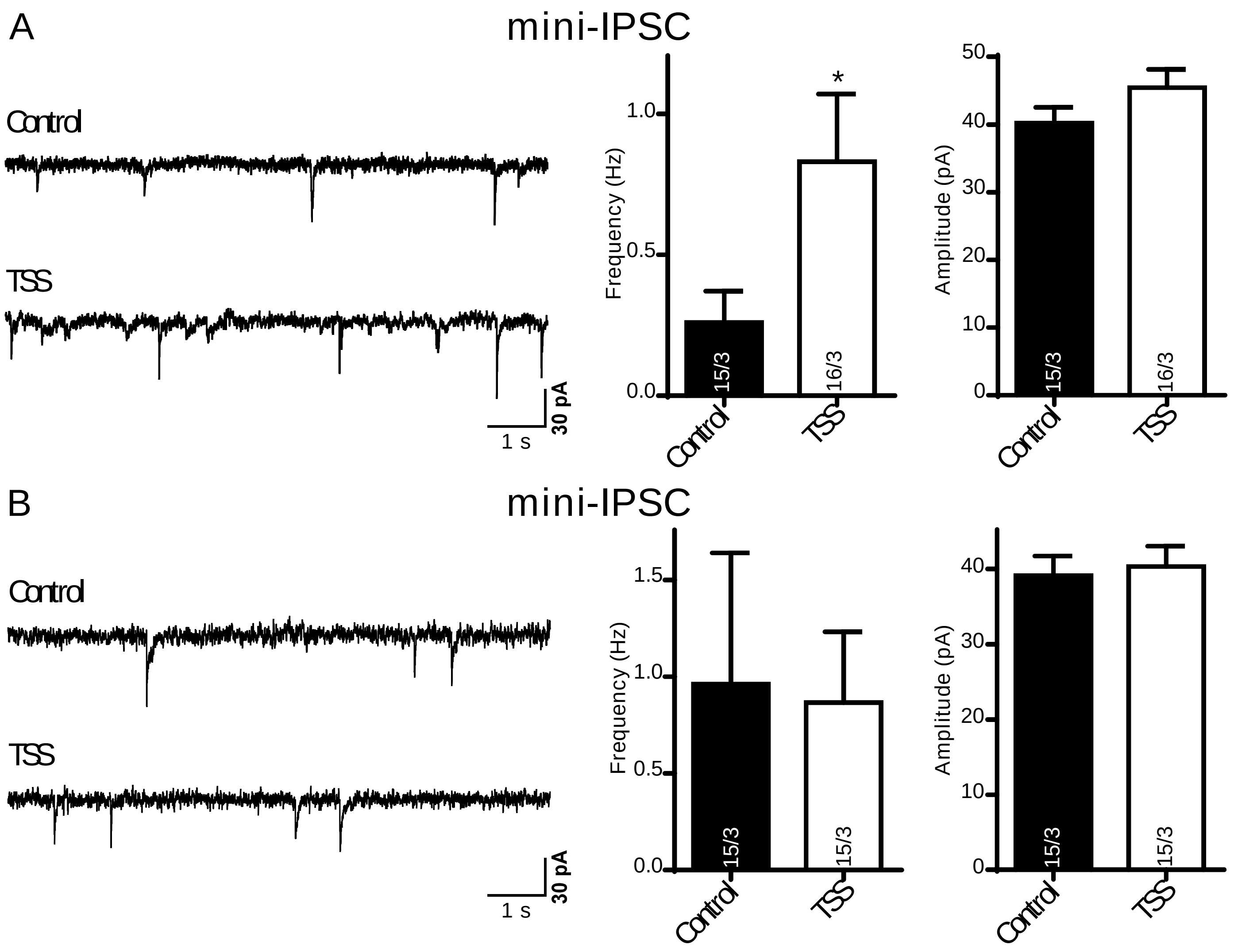
A
mini-IPSC
Control
TSS
30 pA
1 s
Frequency (Hz)
1.0
0.5
0.0
*
15/3
16/3
Control
TSS
Amplitude (pA)
50
40
30
20
10
0
15/3
16/3
Control
TSS
B
mini-IPSC
Control
TSS
30 pA
1 s
Frequency (Hz)
1.5
1.0
0.5
0.0
15/3
15/3
Control
TSS
Amplitude (pA)
40
30
20
10
0
15/3
15/3
Control
TSS

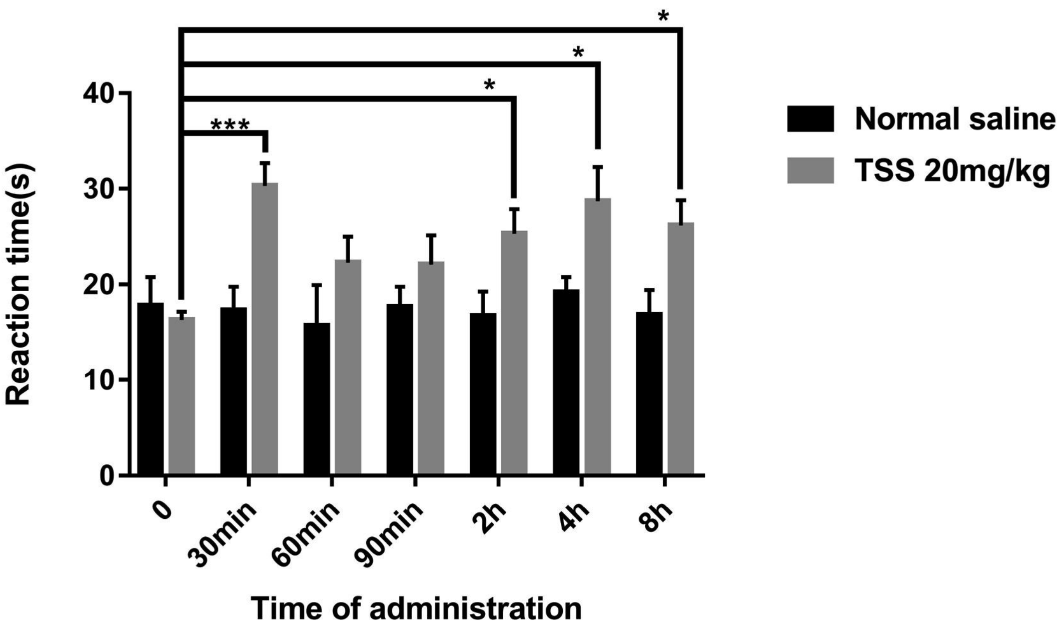

Reaction time(s)
40
30
20
10
0
***
*
*
*
0
30min
60min
90min
2h
4h
8h
Time of administration
Normal saline
TSS 20mg/kg

Electronic Supplementary Information

1. Figure 1: MS spectra of compound **1** page 3
2. Figure 2: MS spectra of compound **2** page 4
3. Figure 3: MS spectra of compound **3** page 5-6
4. Figure 4: MS spectra of compound **4** page 6-7
5. Figure 5: MS spectra of compound **5** page 8
6. Figure 6: MS spectra of compound **6** page 9-10
7. Figure 7: MS spectra of compound **7** page 11-12
8. Figure 8: MS spectra of compound **8** page 13
9. Figure 9: MS spectra of compound **9** page 14
10. Figure 10: MS spectra of compound **10** page 15
11. Figure 11: MS spectra of compound **11** page 16
12. Figure 12: MS spectra of compound **12** page 17-18
13. Figure 13: MS spectra of compound **13** page 19
14. Figure 14: MS spectra of compound **14** page 20
15. Figure 15: MS spectra of compound **15** page 21-22
16. Figure 16: MS spectra of compound **16** page 23
17. Figure 17: MS spectra of compound **17** page 24-25
18. Figure 18: MS spectra of compound **18** page 26
19. Figure 19: MS spectra of compound **19** page 27
20. Figure 20: MS spectra of compound **20** page 28-29
21. Figure 21: MS total ion current (TIC) chromatogram of TSS page 30
22. Table 1: Triterpenoid saponins identified in the TSS by HPLC-ESI-MS/MS

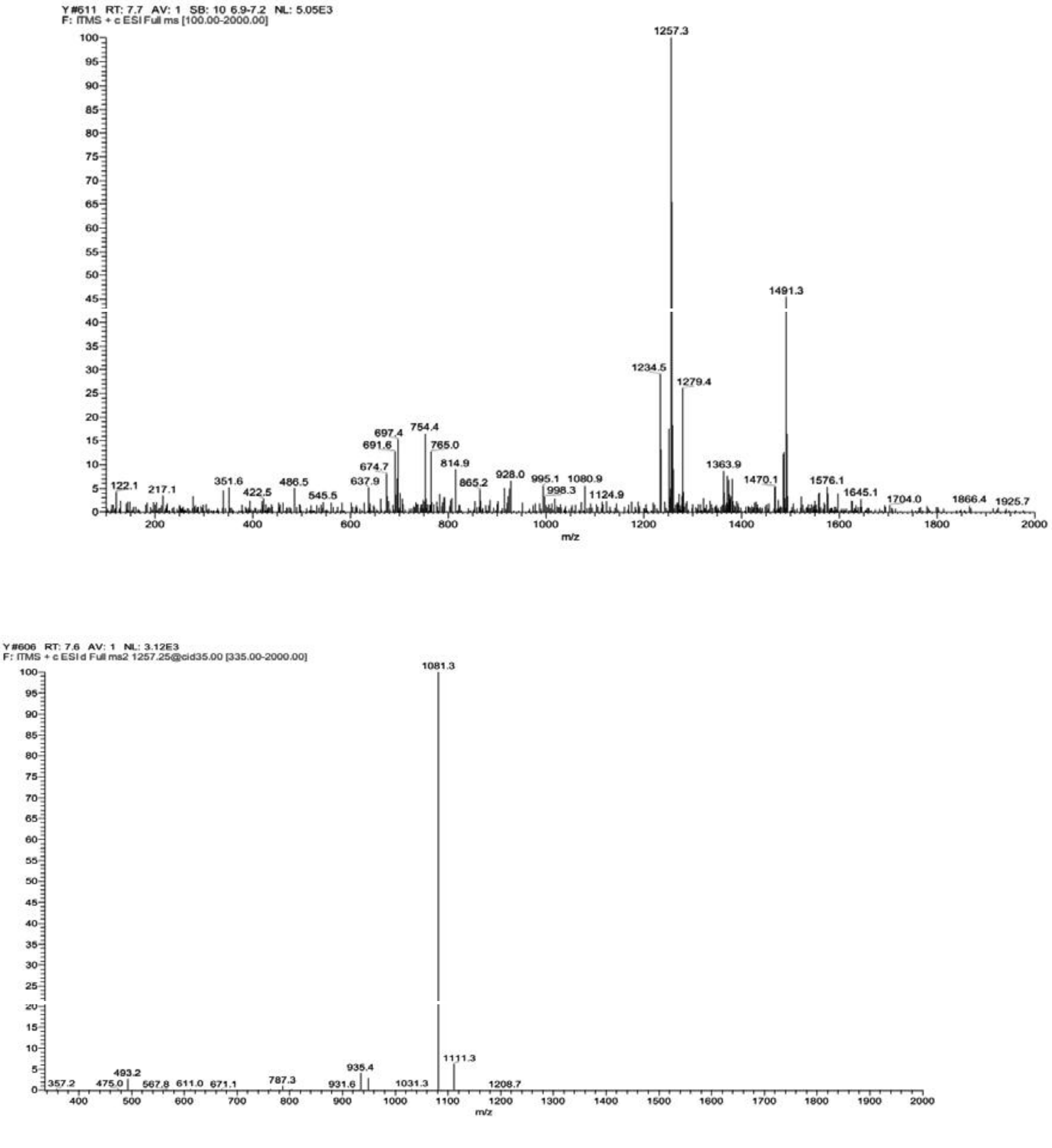

Figure 1: MS spectra of compound **1**

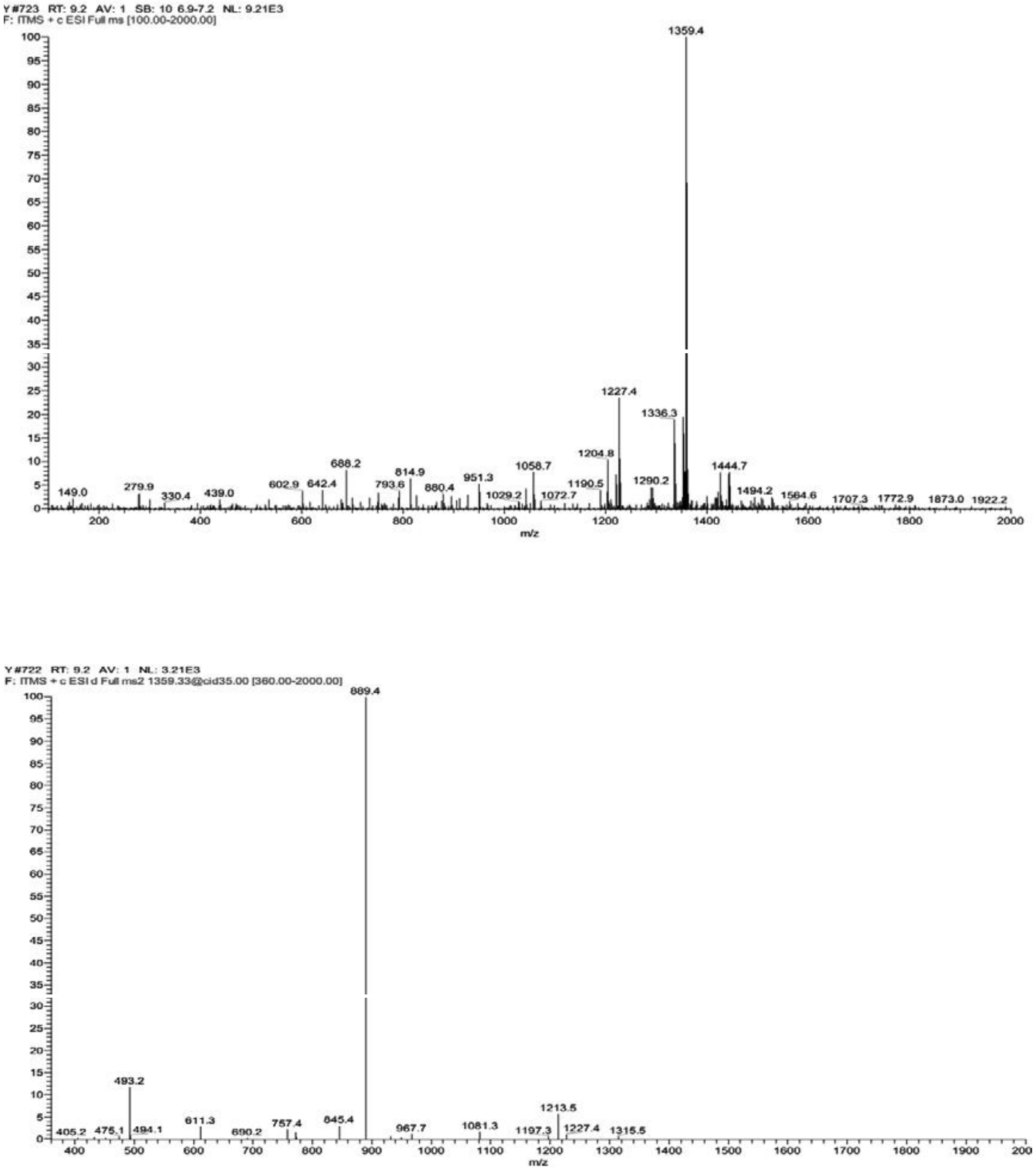

Figure 2: MS spectra of compound **2**

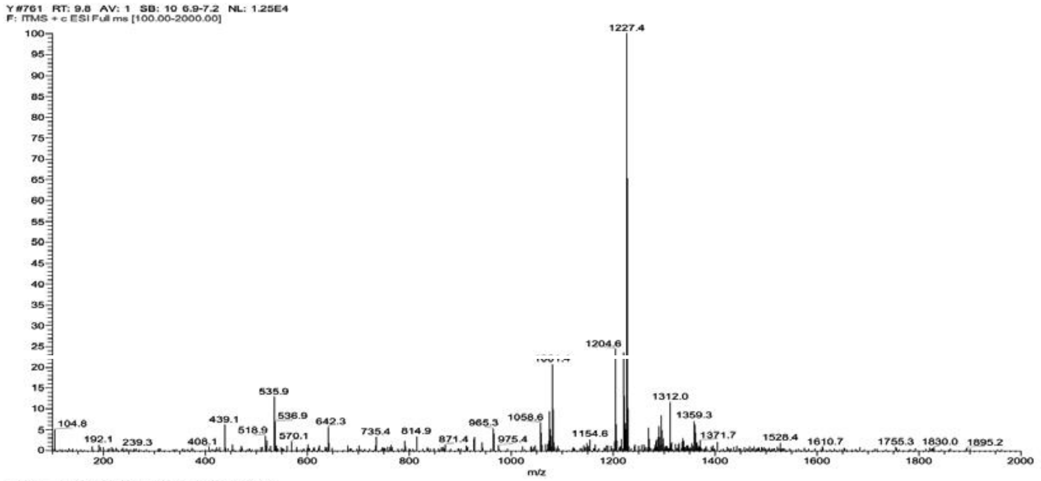
Y #761 RT: 9.8 AV: 1 SB: 10 6.9-7.2 NL: 1.25E4
F: ITMS + c ESI Full ms [100.00-2000.00]
1227.4
1204.6
1312.0
1359.3
1371.7
535.9
536.9
439.1
518.9
570.1
642.3
735.4
814.9
965.3
1058.6
975.4
871.4
1154.6
104.8
192.1
239.3
408.1
1528.4
1610.7
1755.3
1830.0
1895.2
m/z

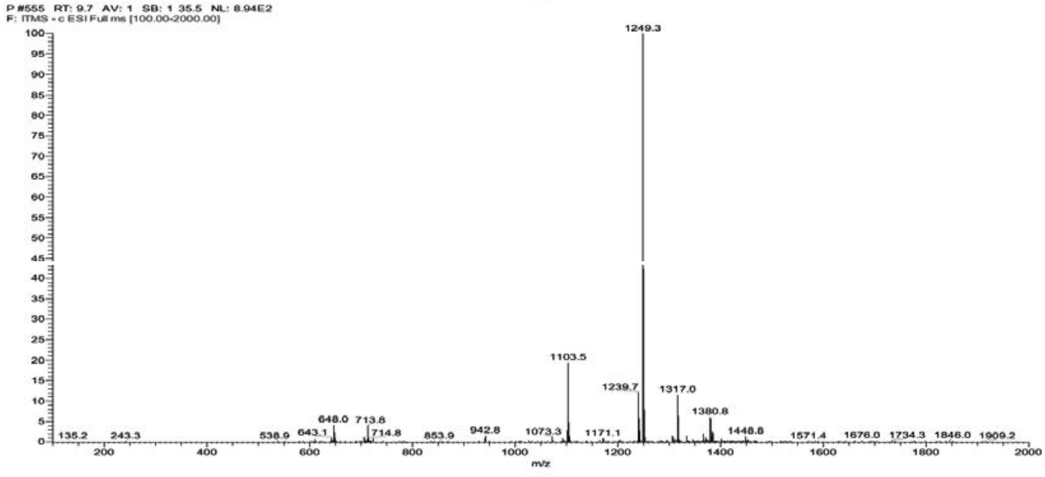
P #555 RT: 9.7 AV: 1 SB: 1 35.5 NL: 8.94E2
F: ITMS + c ESI Full ms [100.00-2000.00]
1249.3
1103.5
1239.7
1317.0
1380.8
1448.8
648.0
713.8
643.1
714.8
538.9
853.9
942.8
1073.3
1171.1
135.2
243.3
1571.4
1676.0
1734.3
1846.0
1909.2
m/z

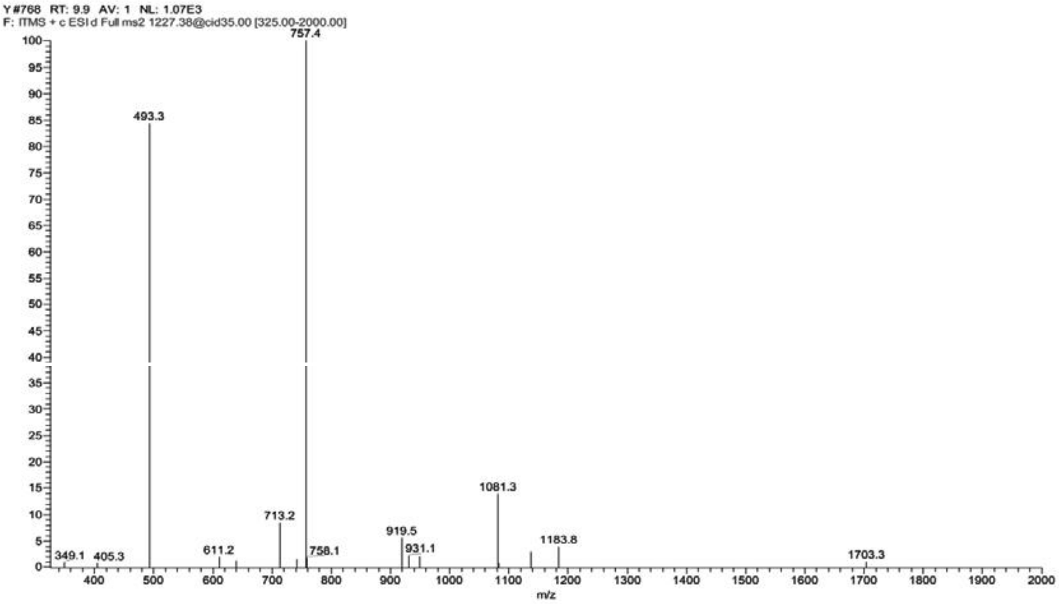

Figure 3: MS spectra of compound **3**

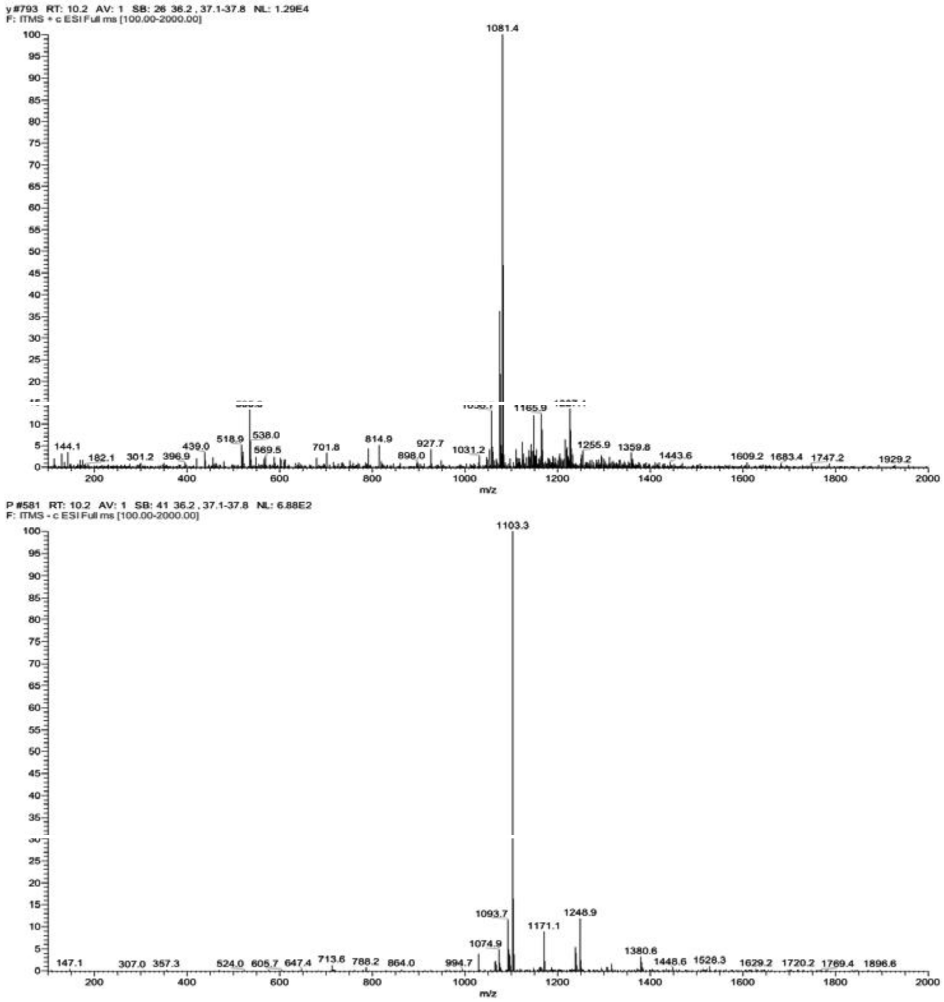

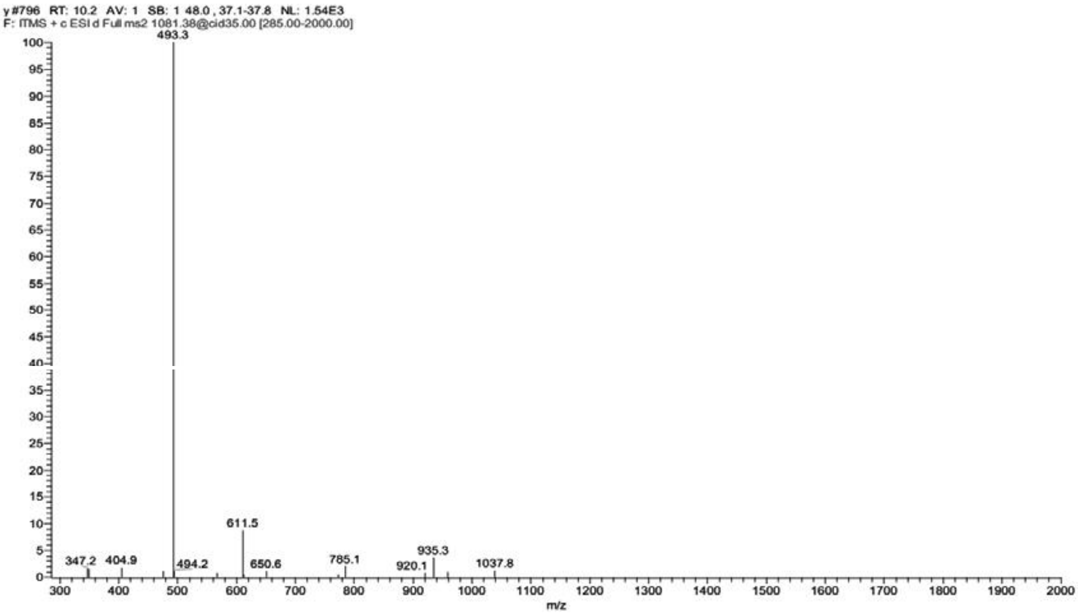

Figure 4: MS spectra of compound **4**

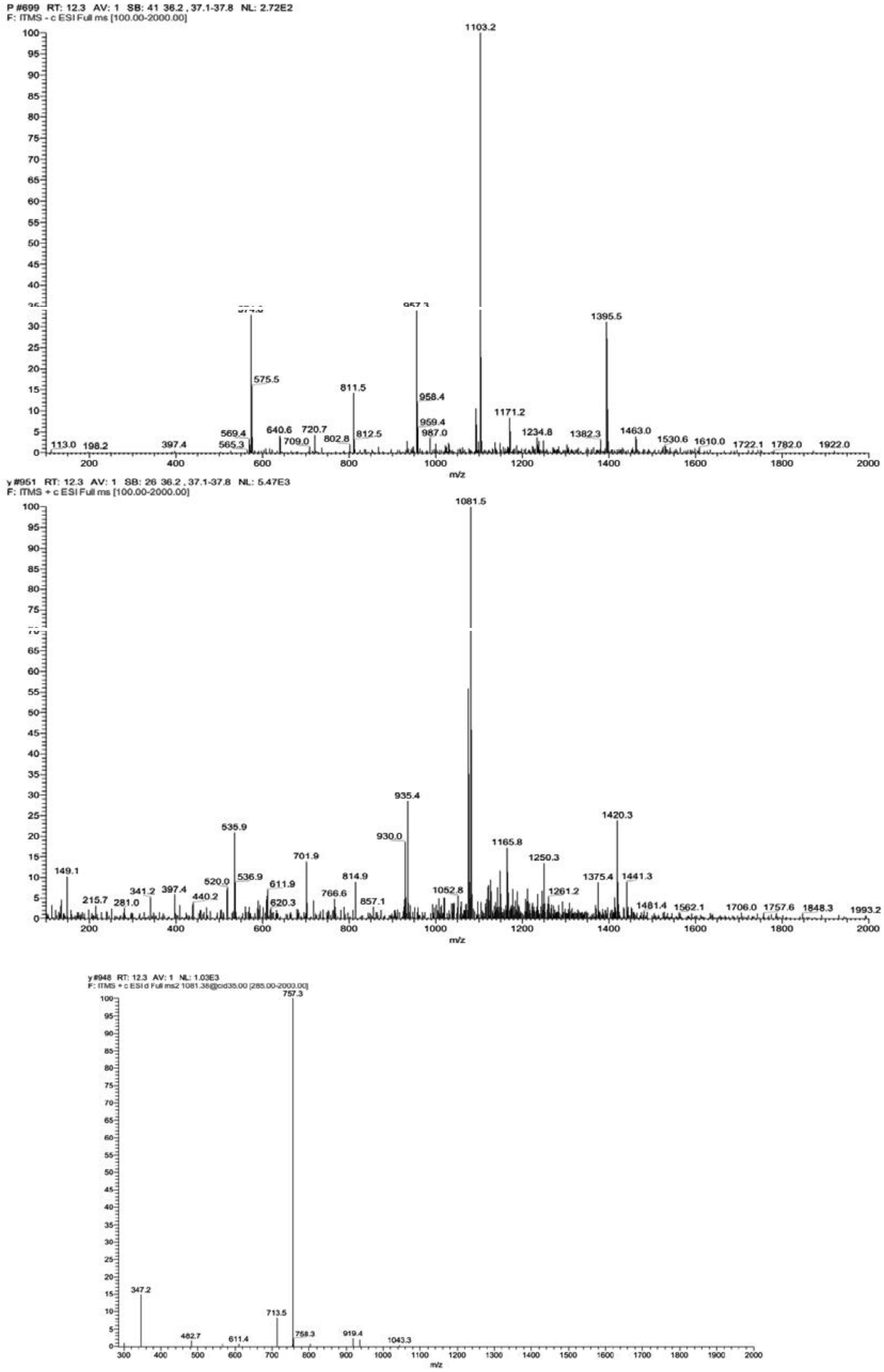

Figure 5: MS spectra of compound **5**

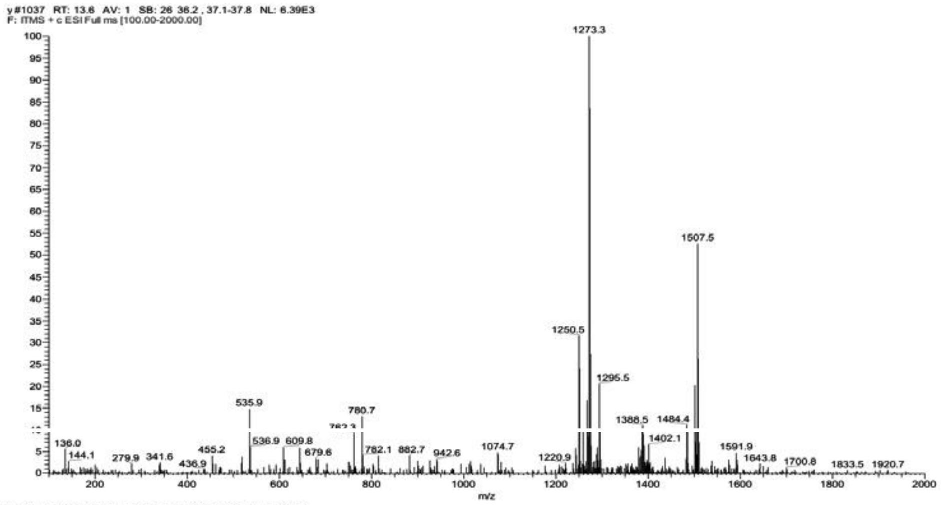
y #1037 RT: 13.6 AV: 1 SB: 26 36.2 , 37.1-37.8 NL: 6.39E3
F: ITMS + c ESI Full ms [100.00-2000.00]
1273.3
1507.5
1250.5
1295.5
535.9
780.7
1388.5
1484.4
1402.1
136.0
144.1
279.9
341.6
436.9
455.2
536.9
609.8
679.6
782.1
882.7
942.6
1074.7
1220.9
1591.9
1643.8
1700.8
1833.5
1920.7
m/z

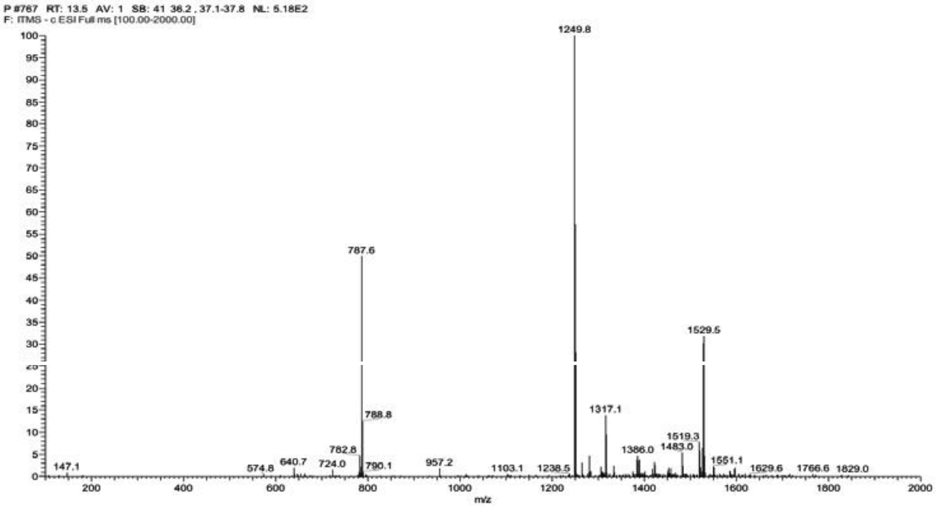
P #767 RT: 13.5 AV: 1 SB: 41 36.2 , 37.1-37.8 NL: 5.18E2
F: ITMS - c ESI Full ms [100.00-2000.00]
1249.8
787.6
1529.5
1317.1
788.8
1519.3
1483.0
1386.0
782.8
147.1
574.8
640.7
724.0
790.1
957.2
1103.1
1238.5
1551.1
1629.6
1766.6
1829.0
m/z

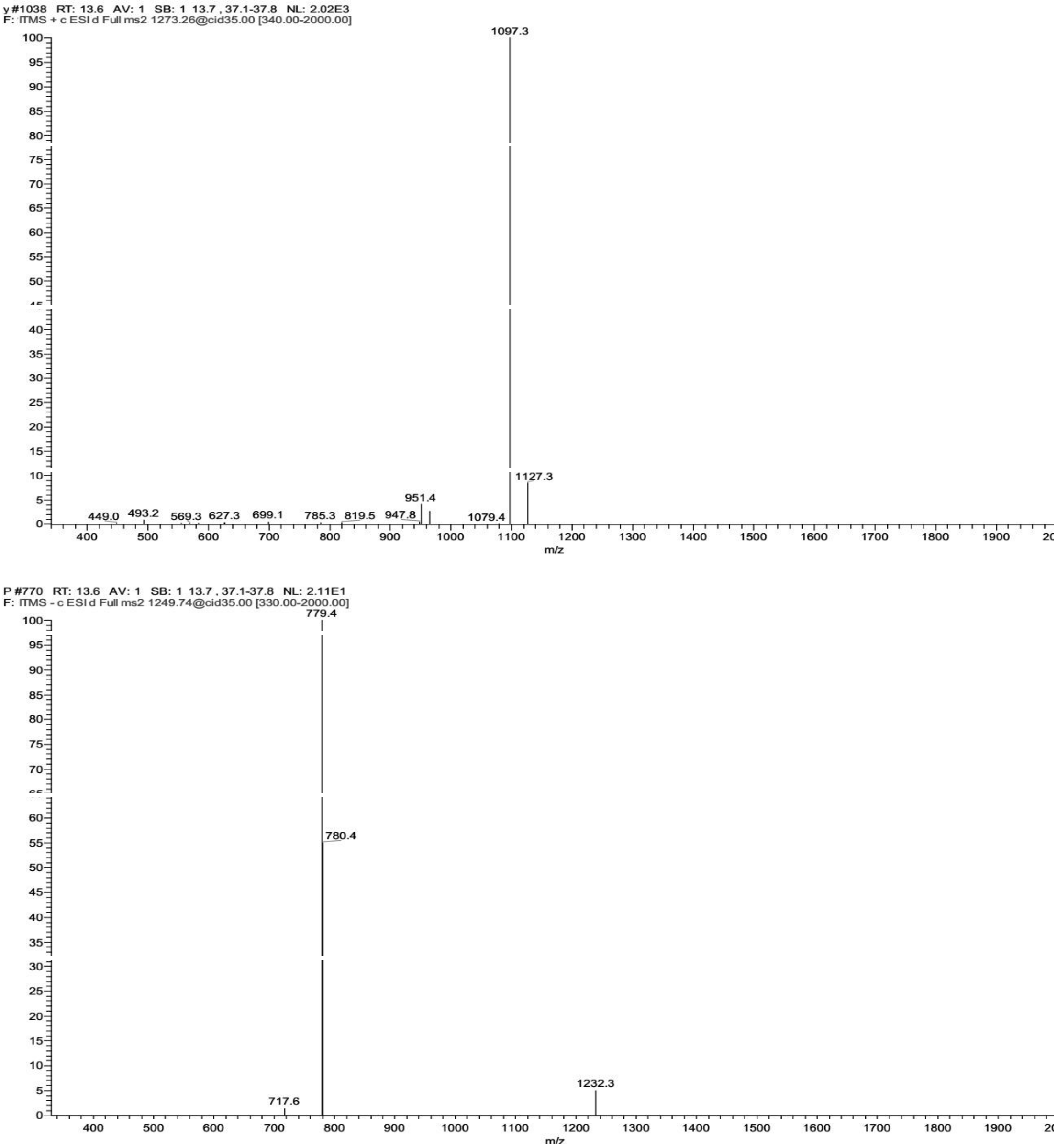

Figure 6: MS spectra of compound **6**

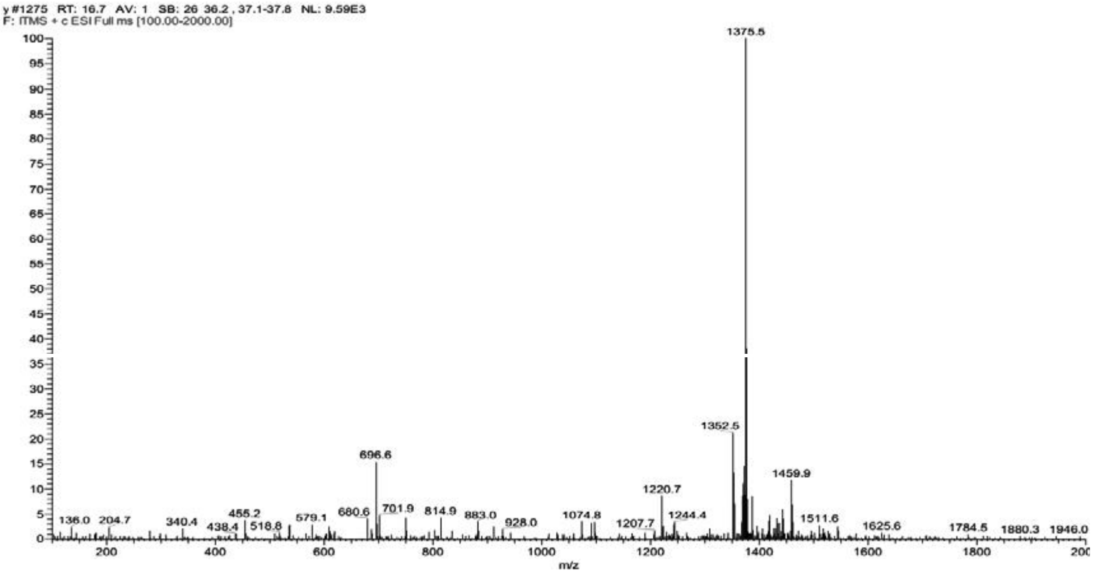
y #1275 RT: 16.7 AV: 1 SB: 26 36.2 , 37.1-37.8 NL: 9.59E3
F: ITMS + c ESI Full ms [100.00-2000.00]
1375.5
1352.5
696.6
1459.9
1220.7
136.0
204.7
340.4
438.4
455.2
518.8
579.1
680.6
701.9
814.9
883.0
928.0
1074.8
1207.7
1244.4
1511.6
1625.6
1784.5
1880.3
1946.0
m/z

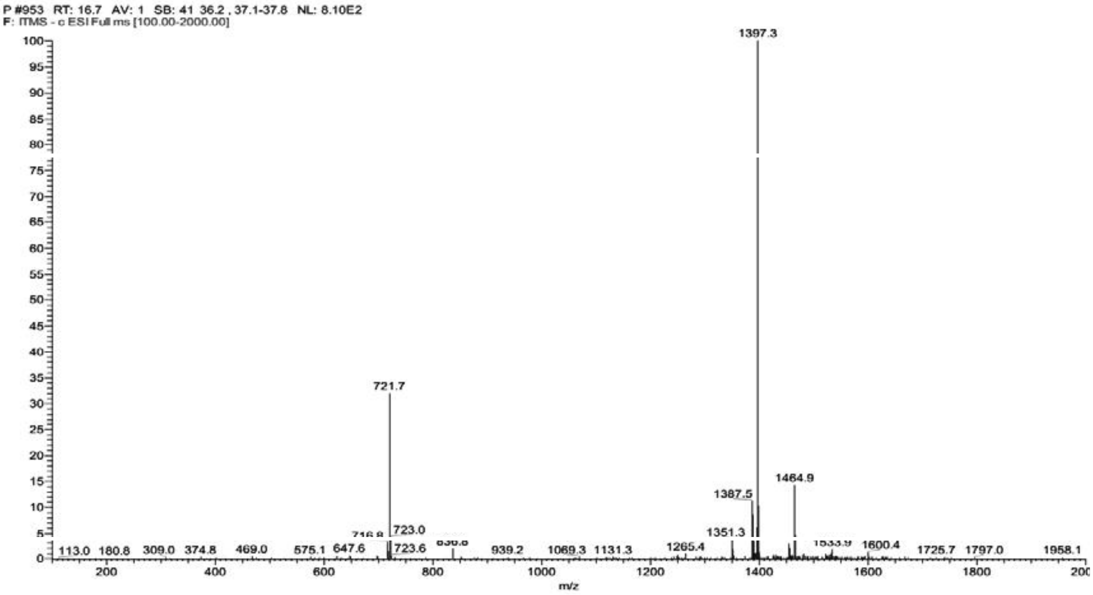
P #953 RT: 16.7 AV: 1 SB: 41 36.2 , 37.1-37.8 NL: 8.10E2
F: ITMS - c ESI Full ms [100.00-2000.00]
1397.3
721.7
1464.9
1387.5
1351.3
113.0
180.8
309.0
374.8
469.0
575.1
647.6
723.0
723.6
939.2
1069.3
1131.3
1265.4
1600.4
1725.7
1797.0
1958.1
m/z

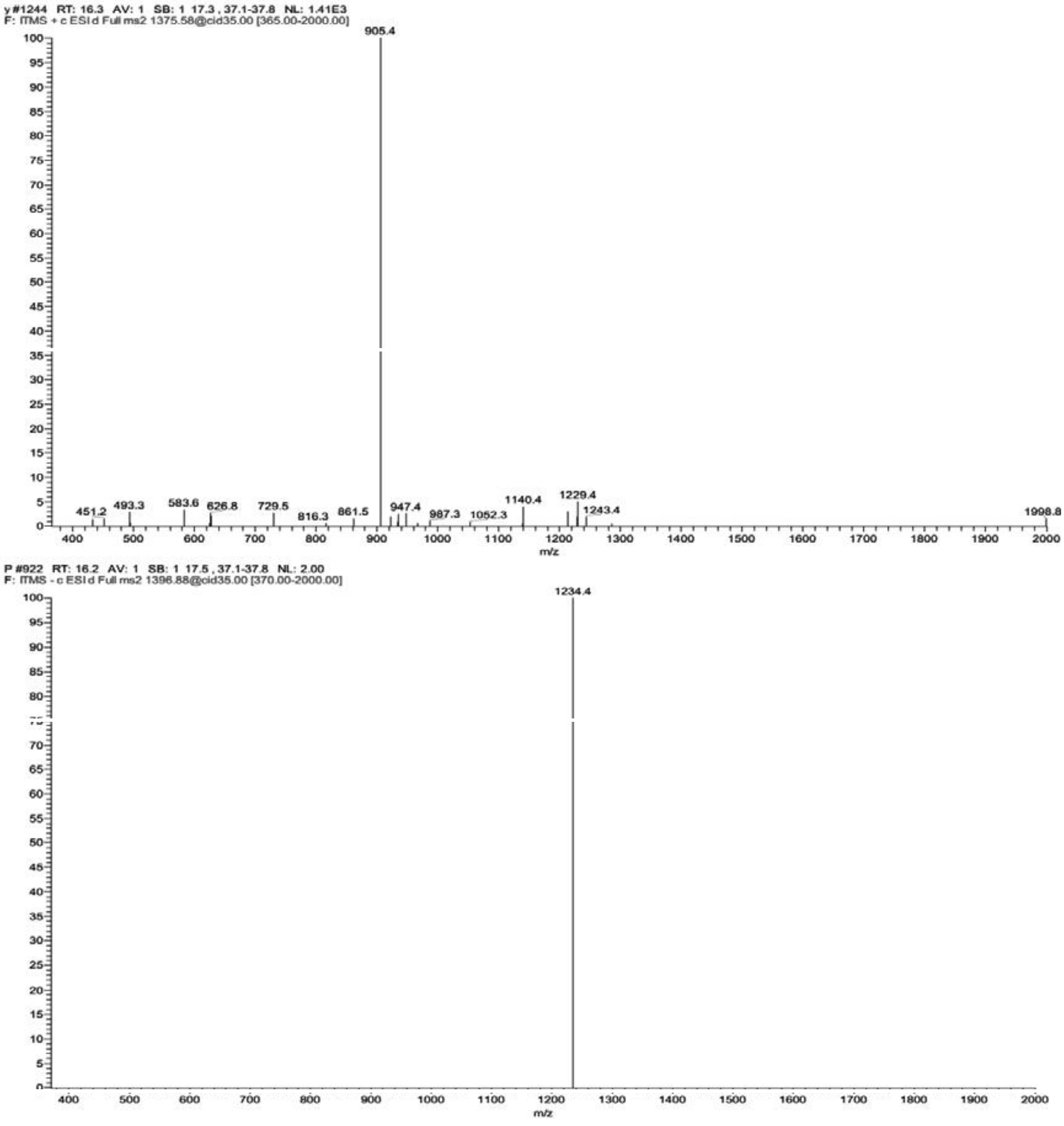

Figure 7: MS spectra of compound **7**

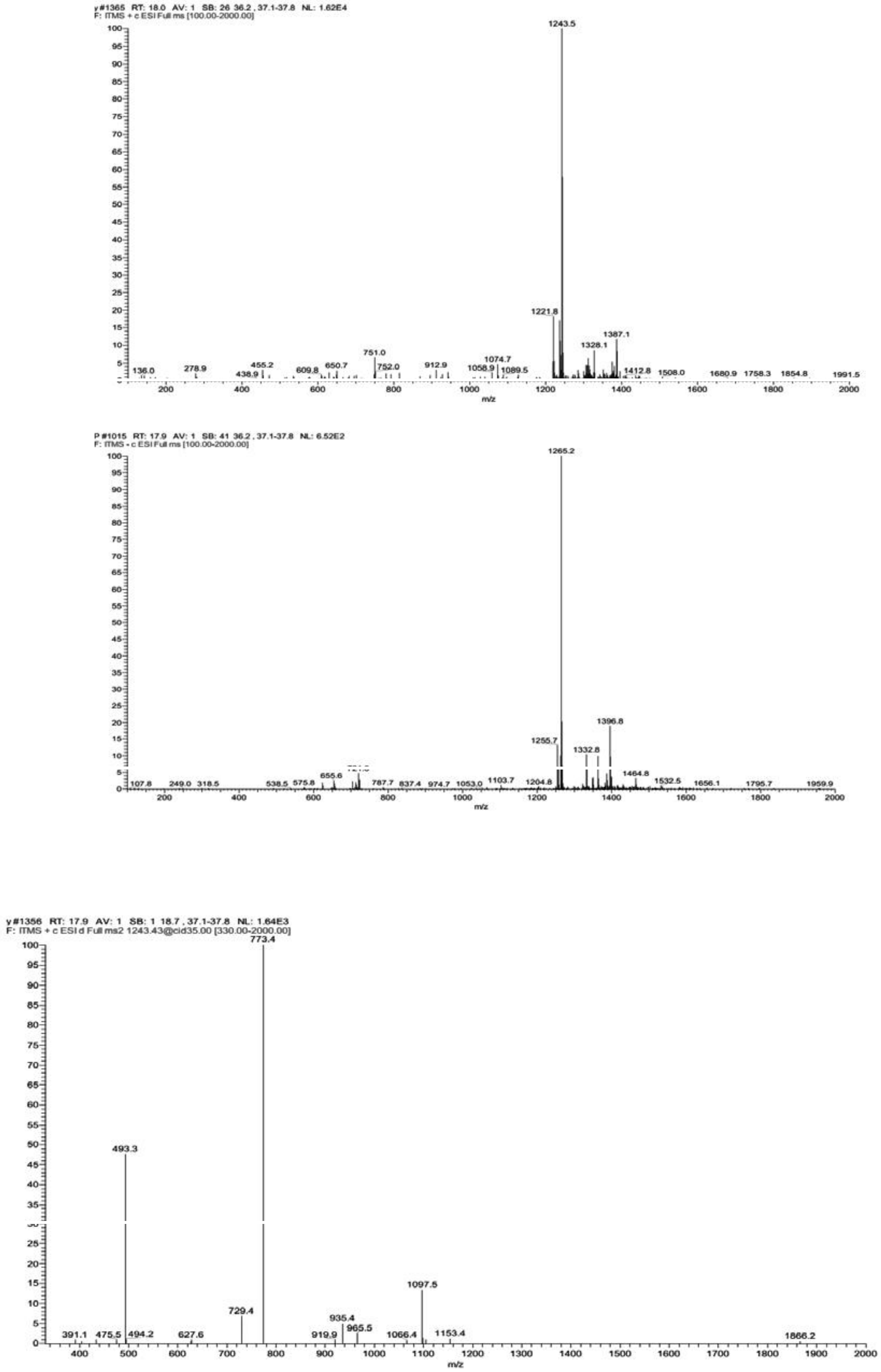

Figure 8: MS spectra of compound **8**

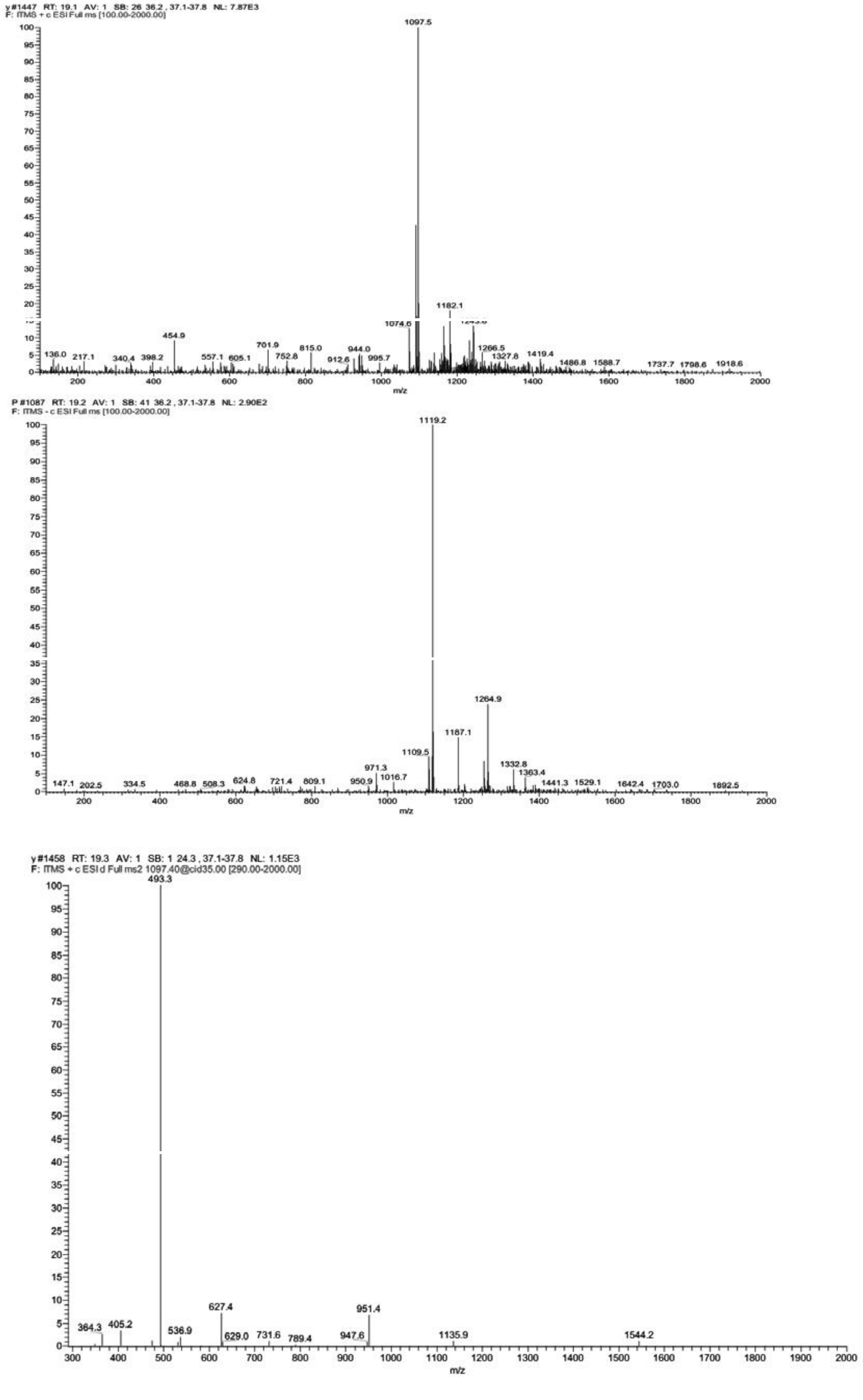

Figure 9: MS spectra of compound **9**

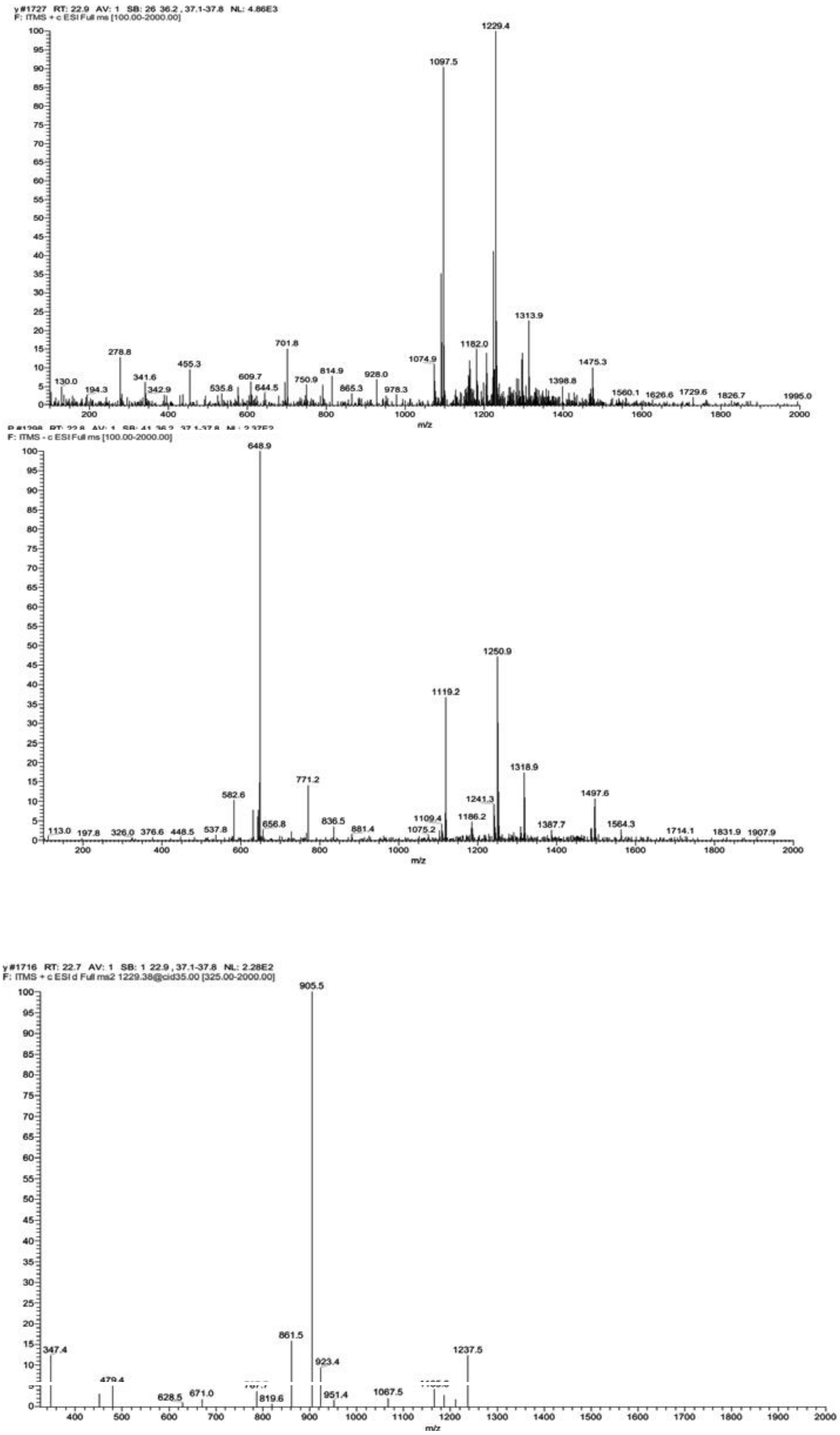

Figure 10: MS spectra of compound **10**

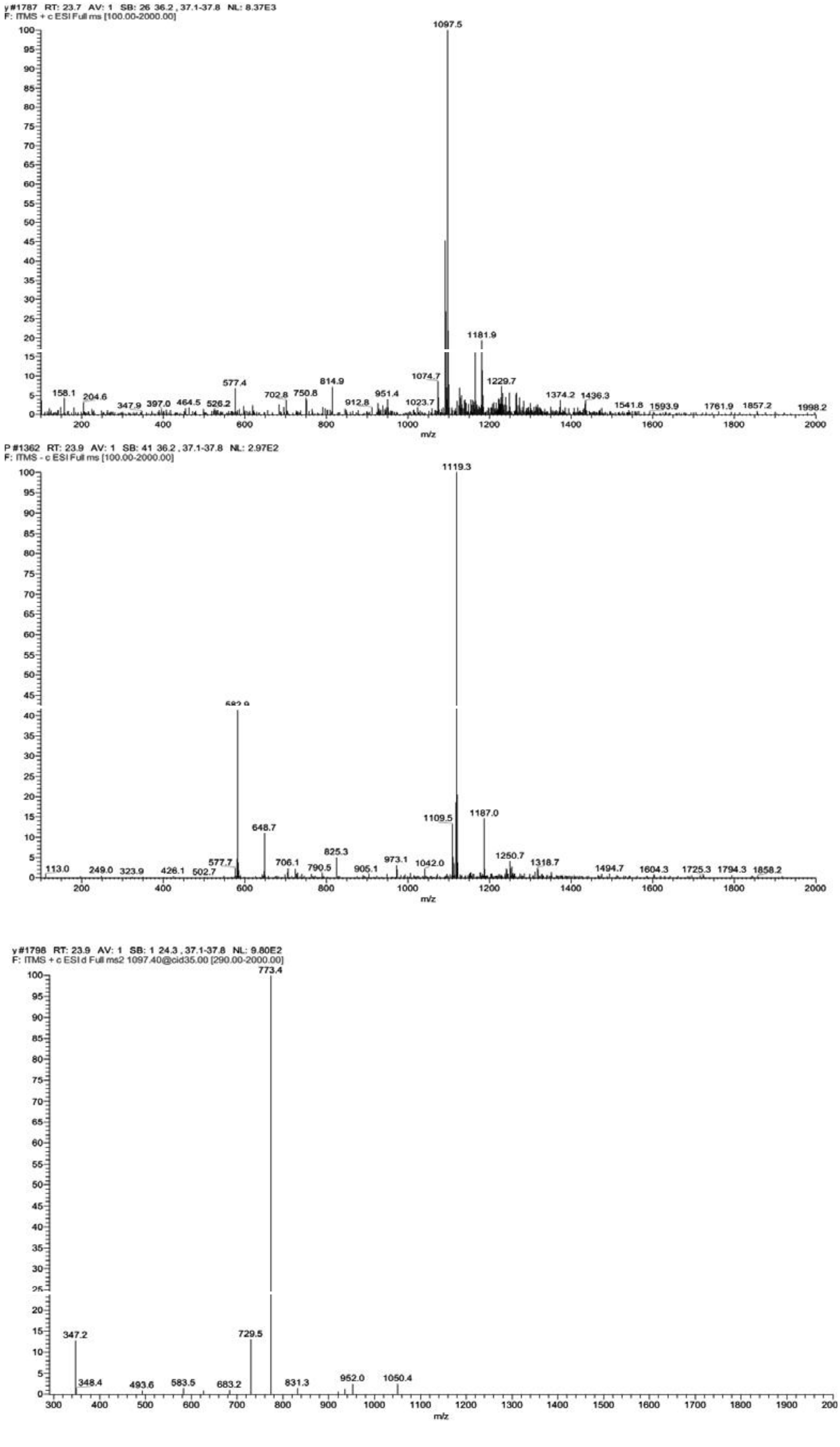

Figure 11: MS spectra of compound **11**

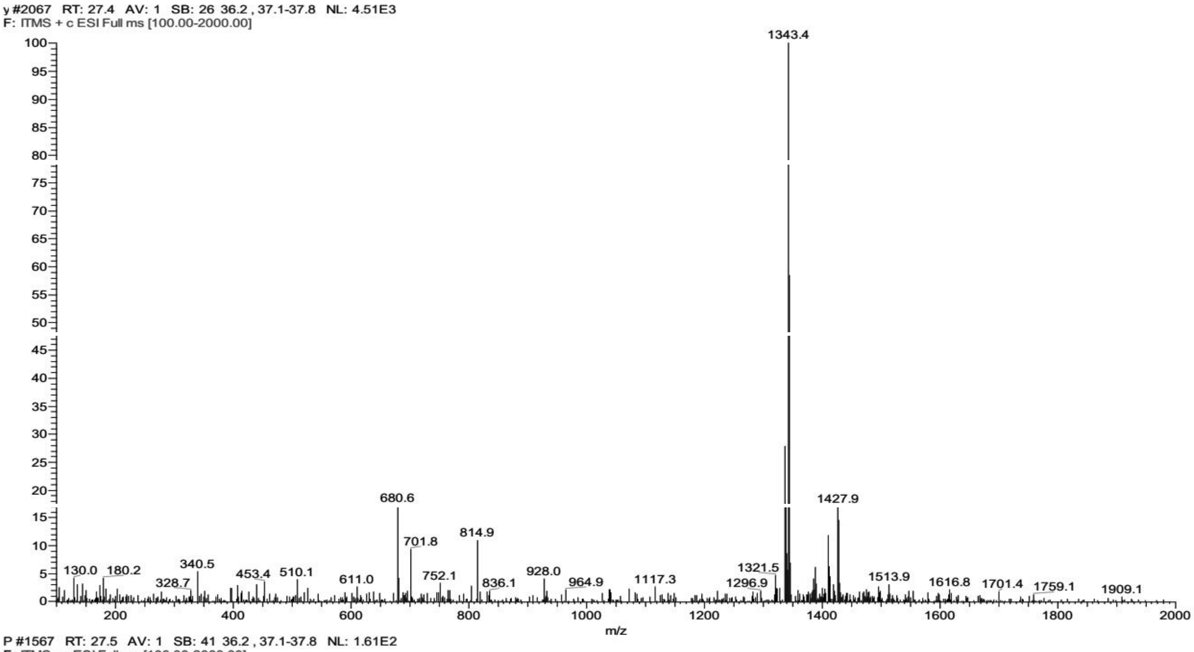
y #2067 RT: 27.4 AV: 1 SB: 26 36.2 , 37.1-37.8 NL: 4.51E3
F: ITMS + c ESI Full ms [100.00-2000.00]
1343.4
680.6
1427.9
814.9
701.8
m/z

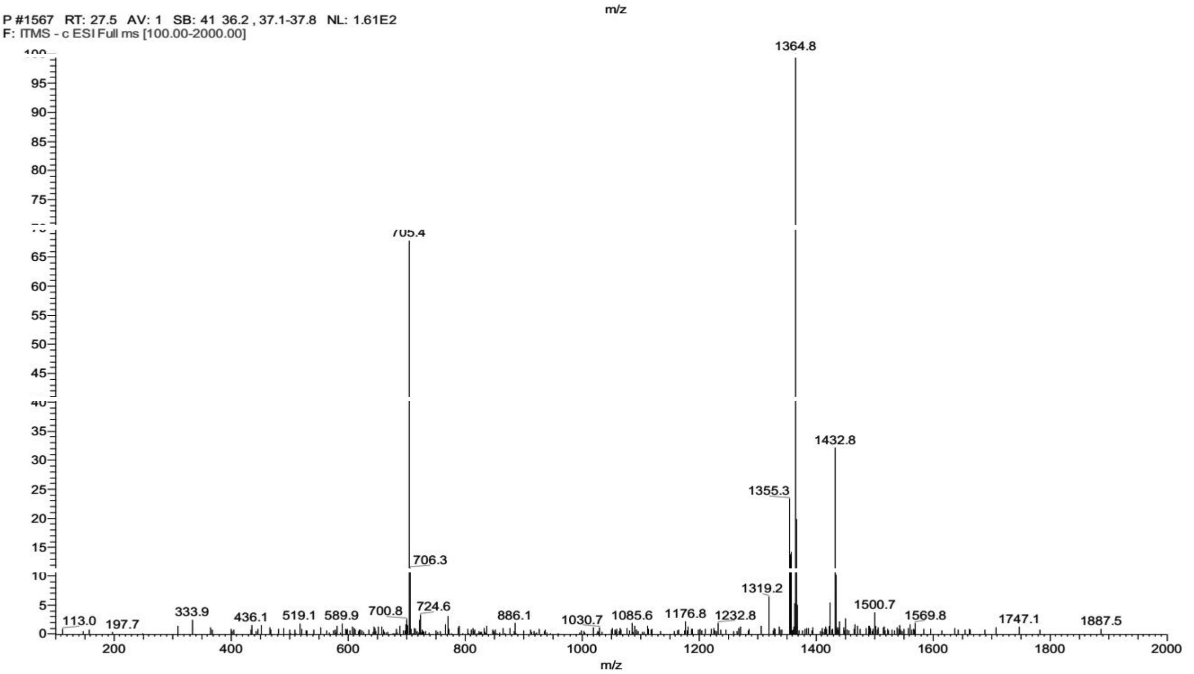
P #1567 RT: 27.5 AV: 1 SB: 41 36.2 , 37.1-37.8 NL: 1.61E2
F: ITMS - c ESI Full ms [100.00-2000.00]
1364.8
705.4
1432.8
1355.3
706.3
m/z

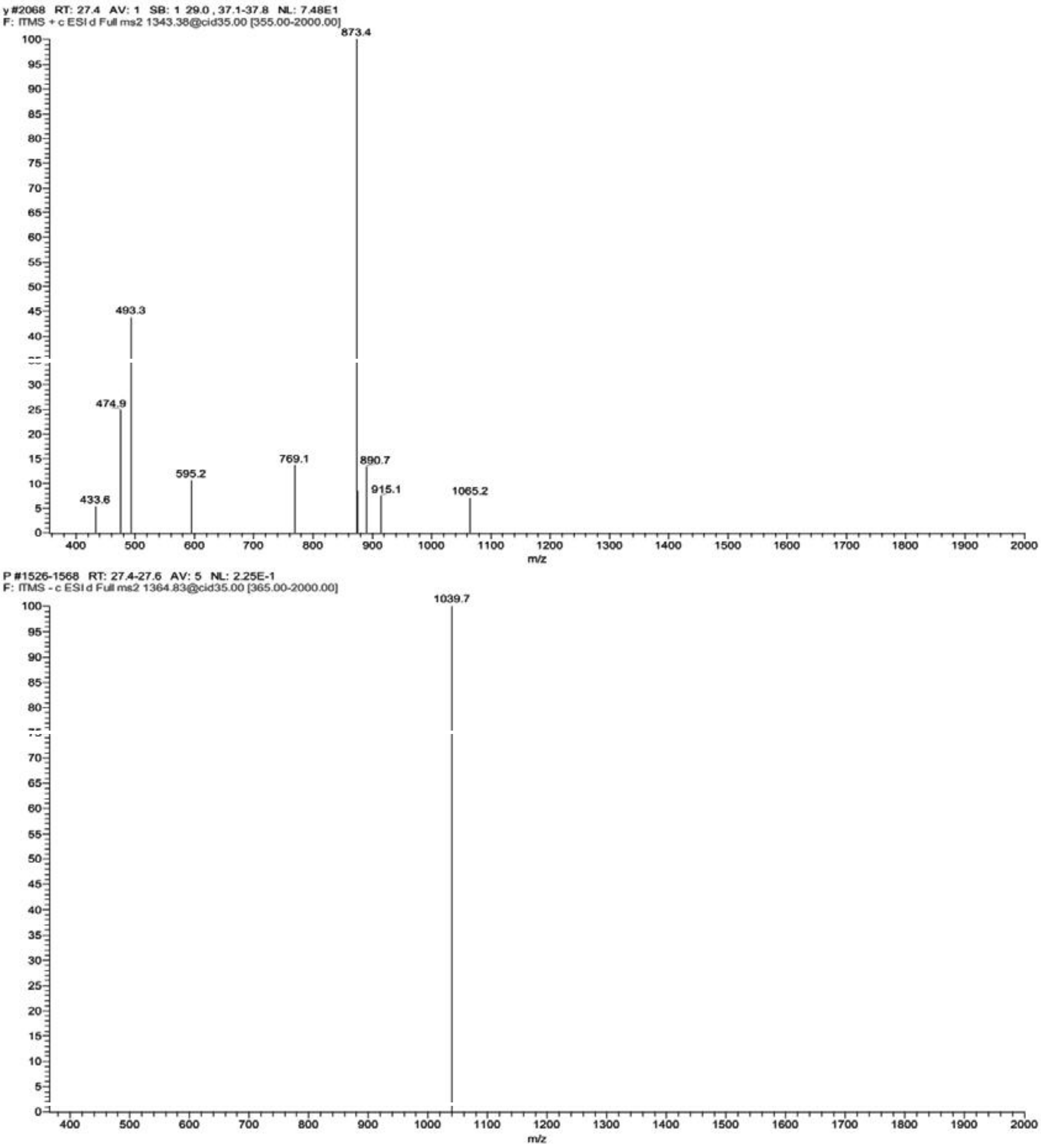

Figure 12: MS spectra of compound **12**

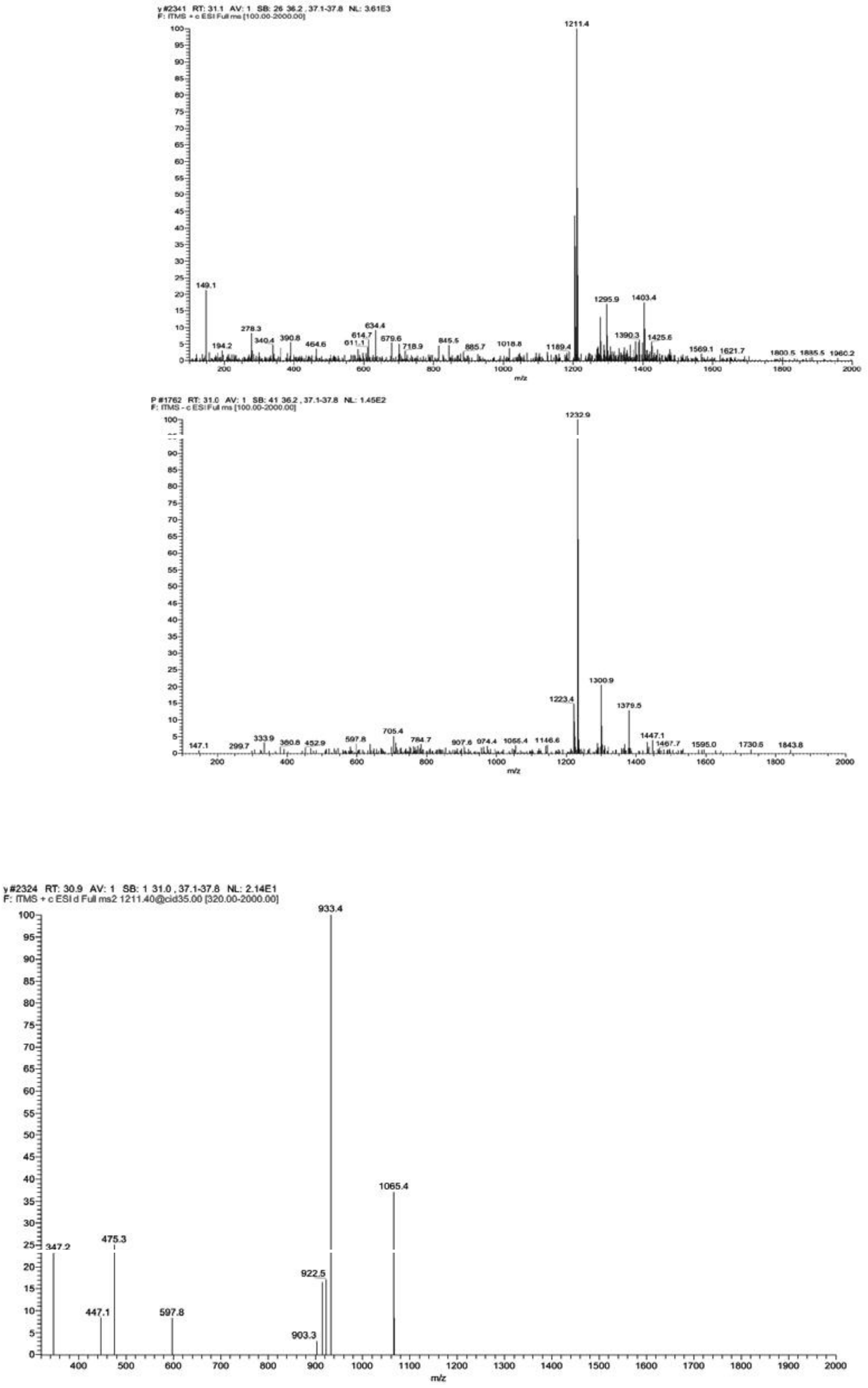

Figure 13: MS spectra of compound **13**

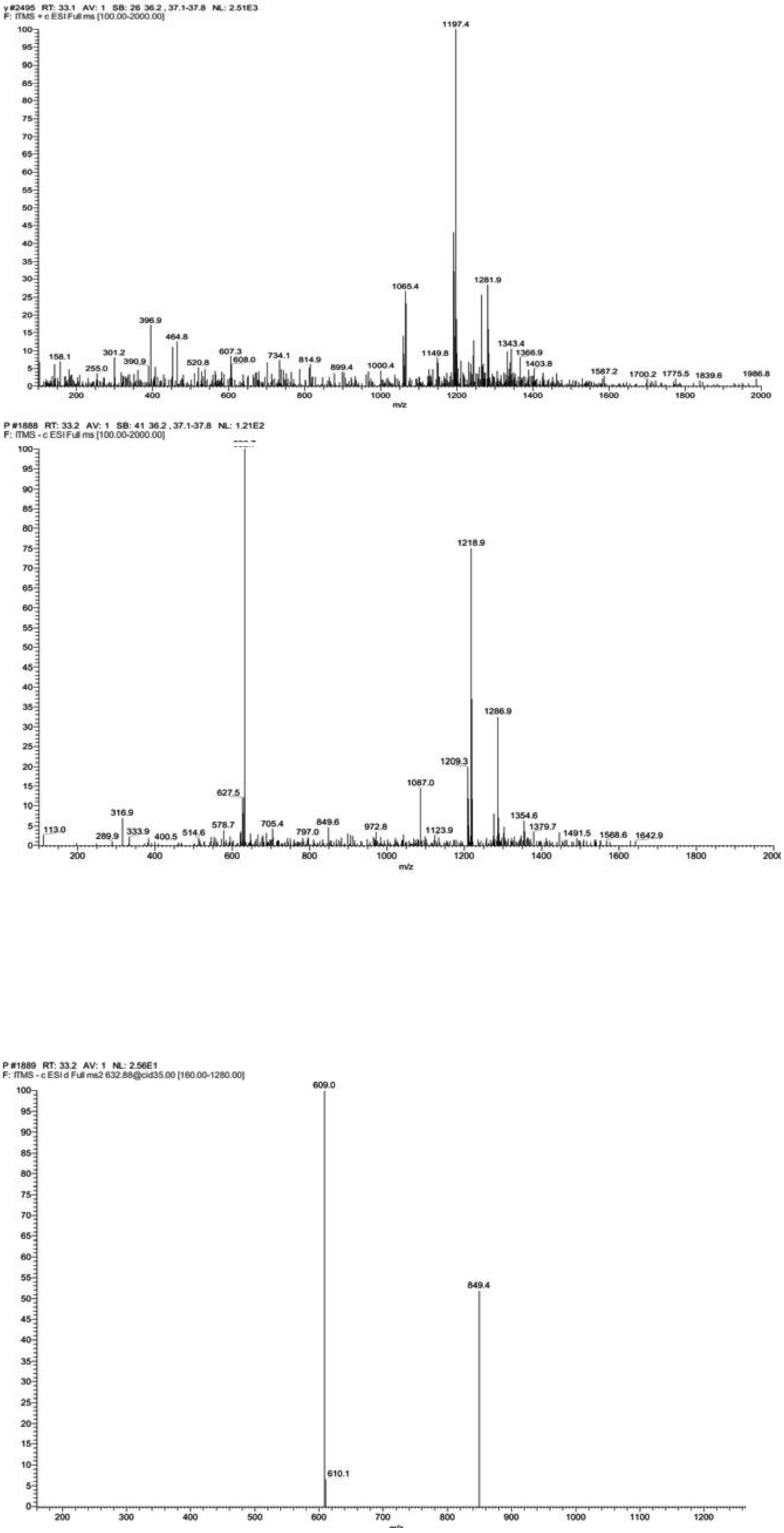

Figure 14: MS spectra of compound **14**

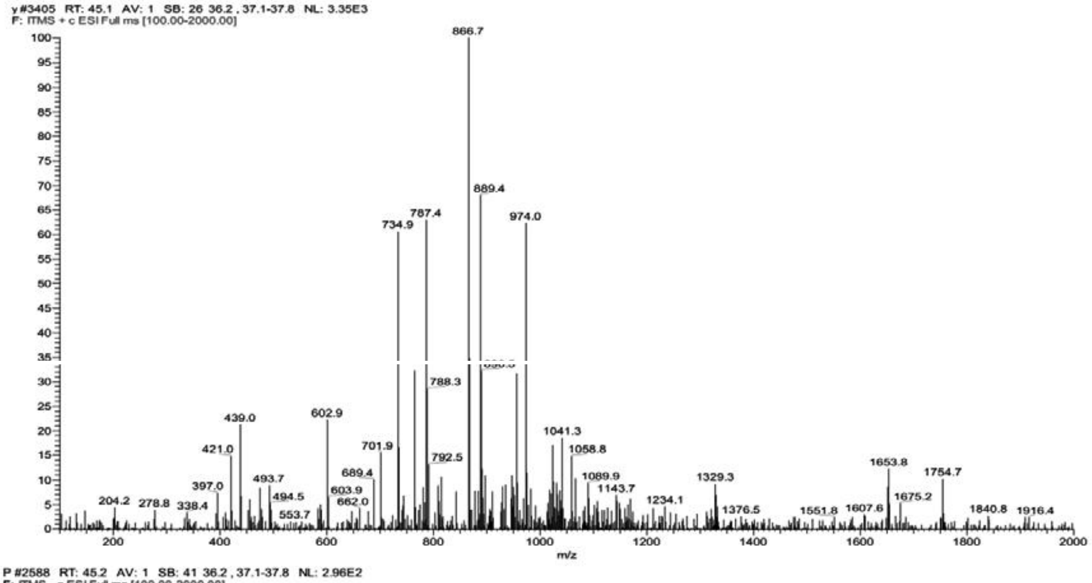
y #3405 RT: 45.1 AV: 1 SB: 26 36.2 , 37.1-37.8 NL: 3.35E3
F: ITMS + c ESI Full ms [100.00-2000.00]
866.7
889.4
787.4
734.9
974.0
788.3
439.0
602.9
1041.3
421.0
701.9
1058.8
689.4
792.5
1653.8
1754.7
397.0
493.7
1089.9
1143.7
1329.3
494.5
603.9
204.2
278.8
338.4
553.7
662.0
1234.1
1376.5
1551.8
1607.6
1675.2
1840.8
1916.4
m/z

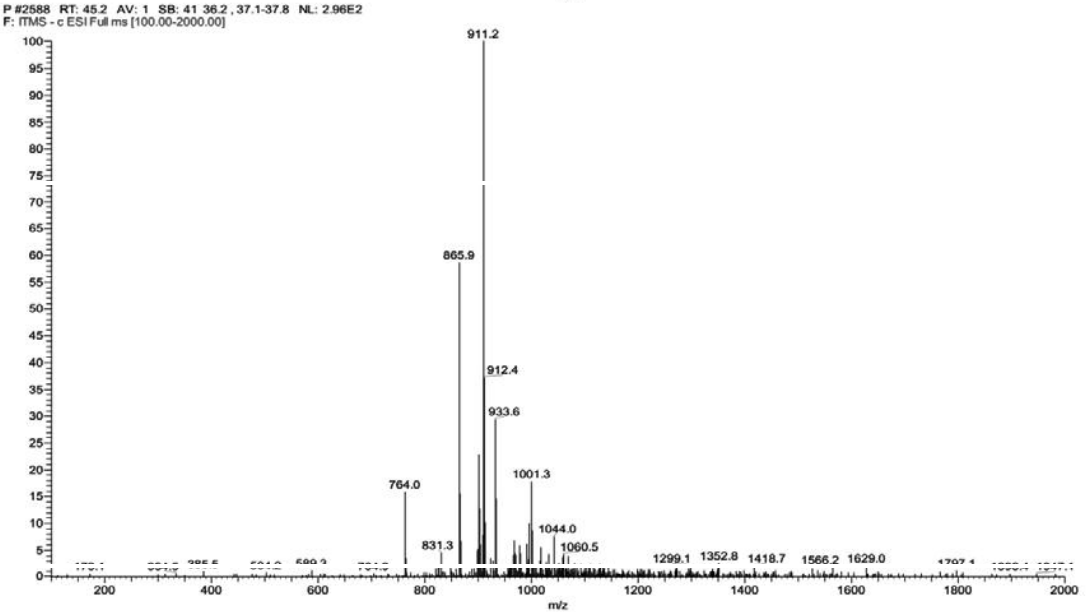
P #2588 RT: 45.2 AV: 1 SB: 41 36.2 , 37.1-37.8 NL: 2.96E2
F: ITMS - c ESI Full ms [100.00-2000.00]
911.2
865.9
912.4
933.6
1001.3
764.0
1044.0
831.3
1060.5
1299.1
1352.8
1418.7
1566.2
1629.0
m/z

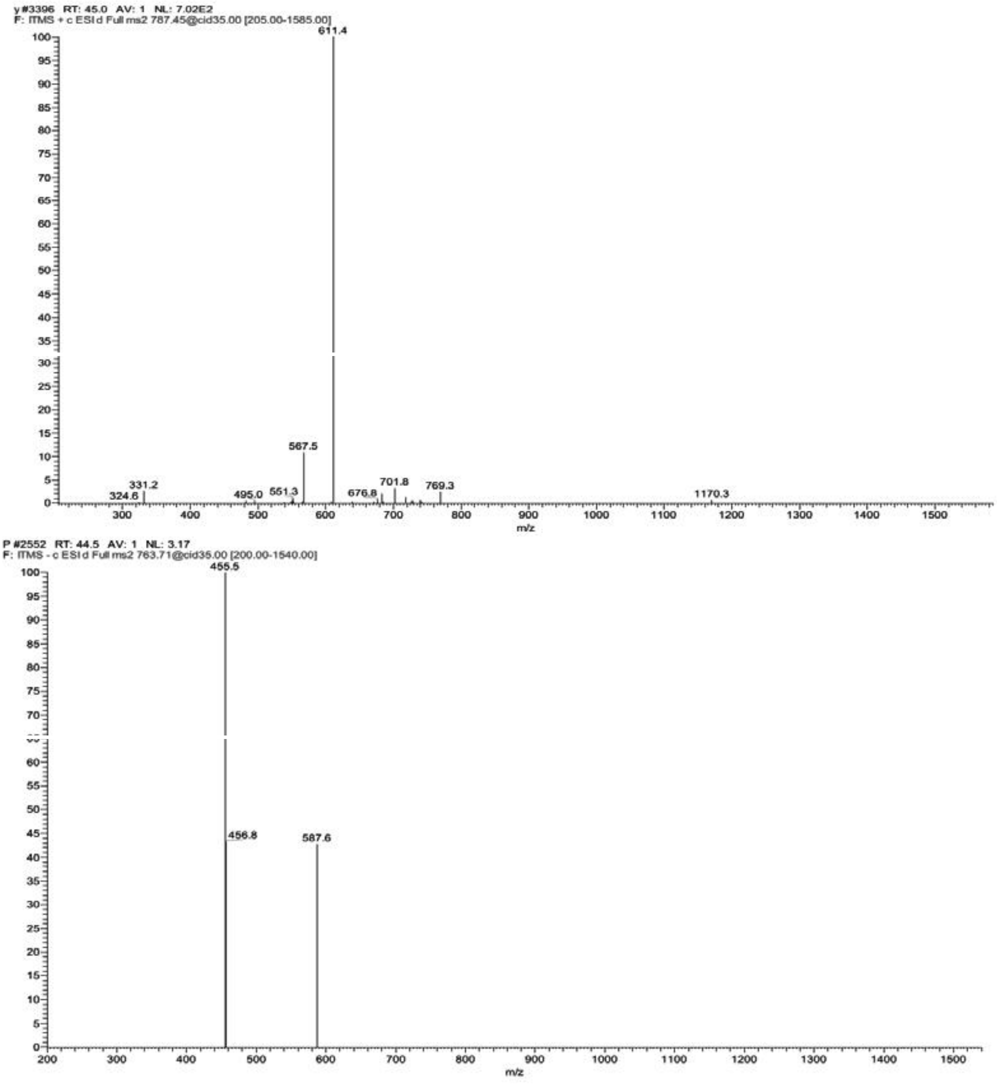

Figure 15: MS spectra of compound **15**

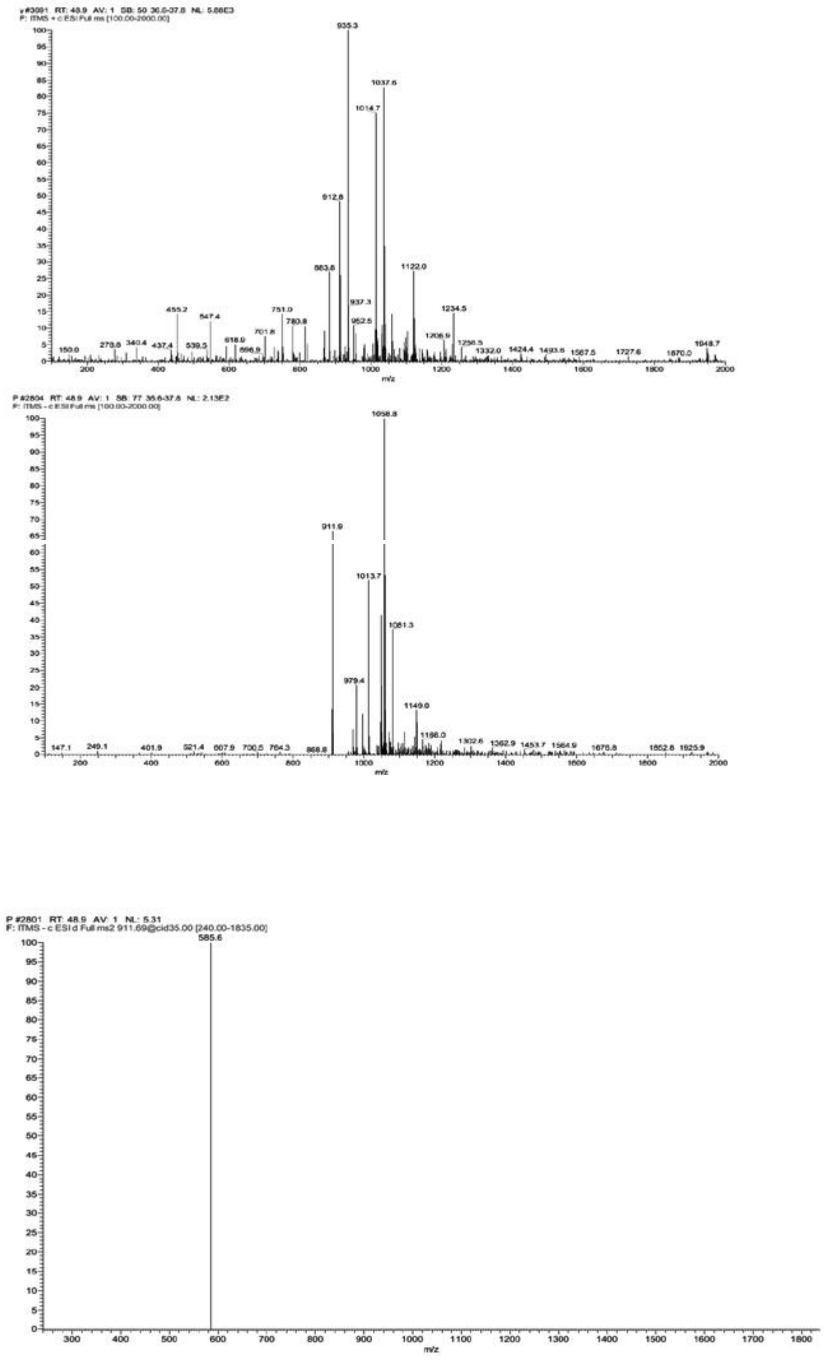

Figure 16: MS spectra of compound **16**

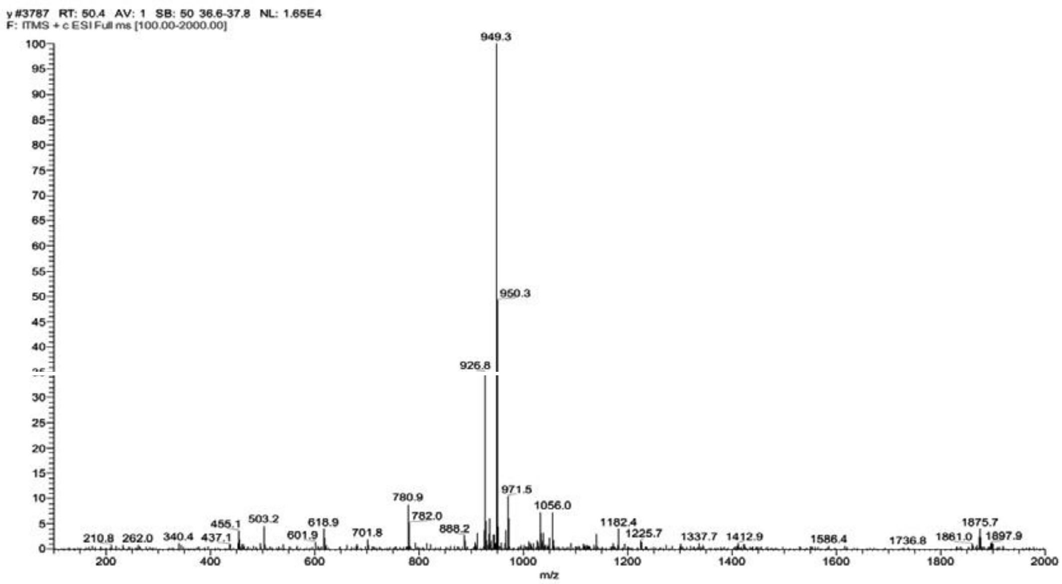
y #3787 RT: 50.4 AV: 1 SB: 50 36.6-37.8 NL: 1.65E4
F: ITMS + c ESI Full ms [100.00-2000.00]
949.3
950.3
926.8
971.5
1056.0
780.9
782.0
888.2
1182.4
1225.7
1337.7
1412.9
1586.4
1736.8
1861.0
1875.7
1897.9
701.8
618.9
601.9
503.2
455.1
437.1
340.4
262.0
210.8
m/z

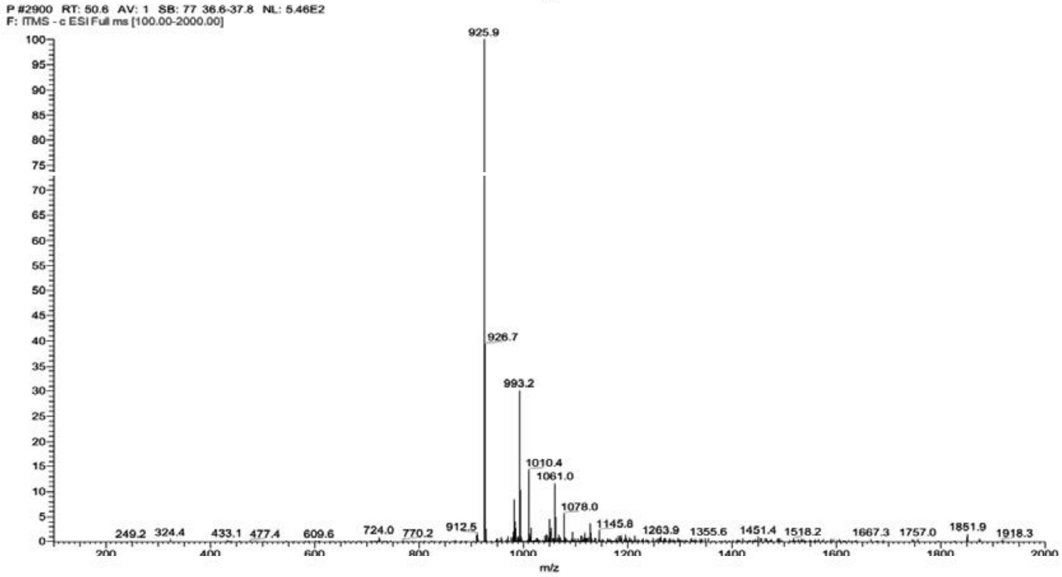
P #2900 RT: 50.6 AV: 1 SB: 77 36.6-37.8 NL: 5.46E2
F: ITMS - c ESI Full ms [100.00-2000.00]
925.9
926.7
993.2
1010.4
1061.0
1078.0
1145.8
1263.9
1355.6
1451.4
1518.2
1667.3
1757.0
1851.9
1918.3
912.5
770.2
724.0
609.6
477.4
433.1
324.4
249.2
m/z

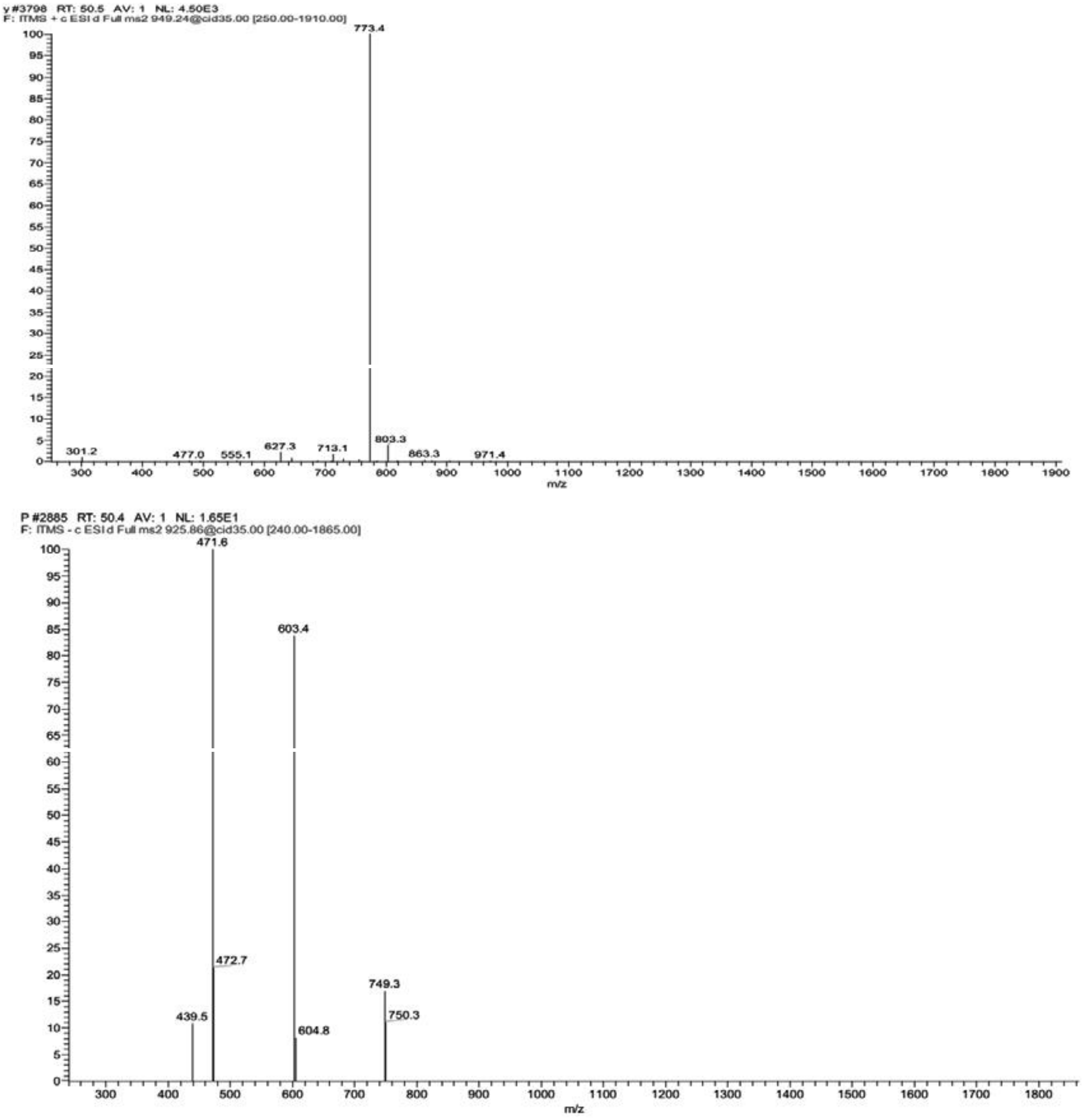

Figure 17: MS spectra of compound **17**

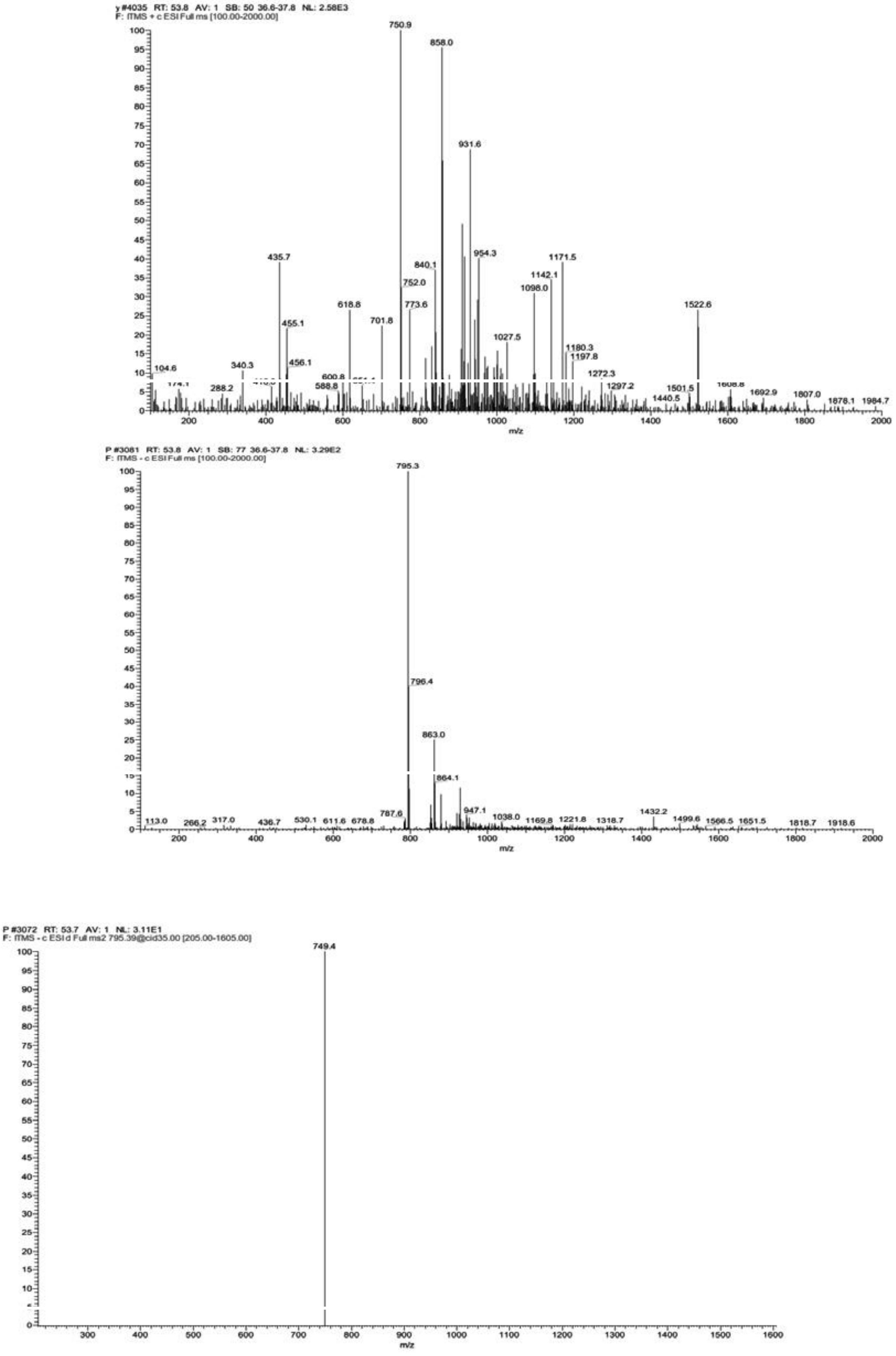

Figure 18: MS spectra of compound **18**

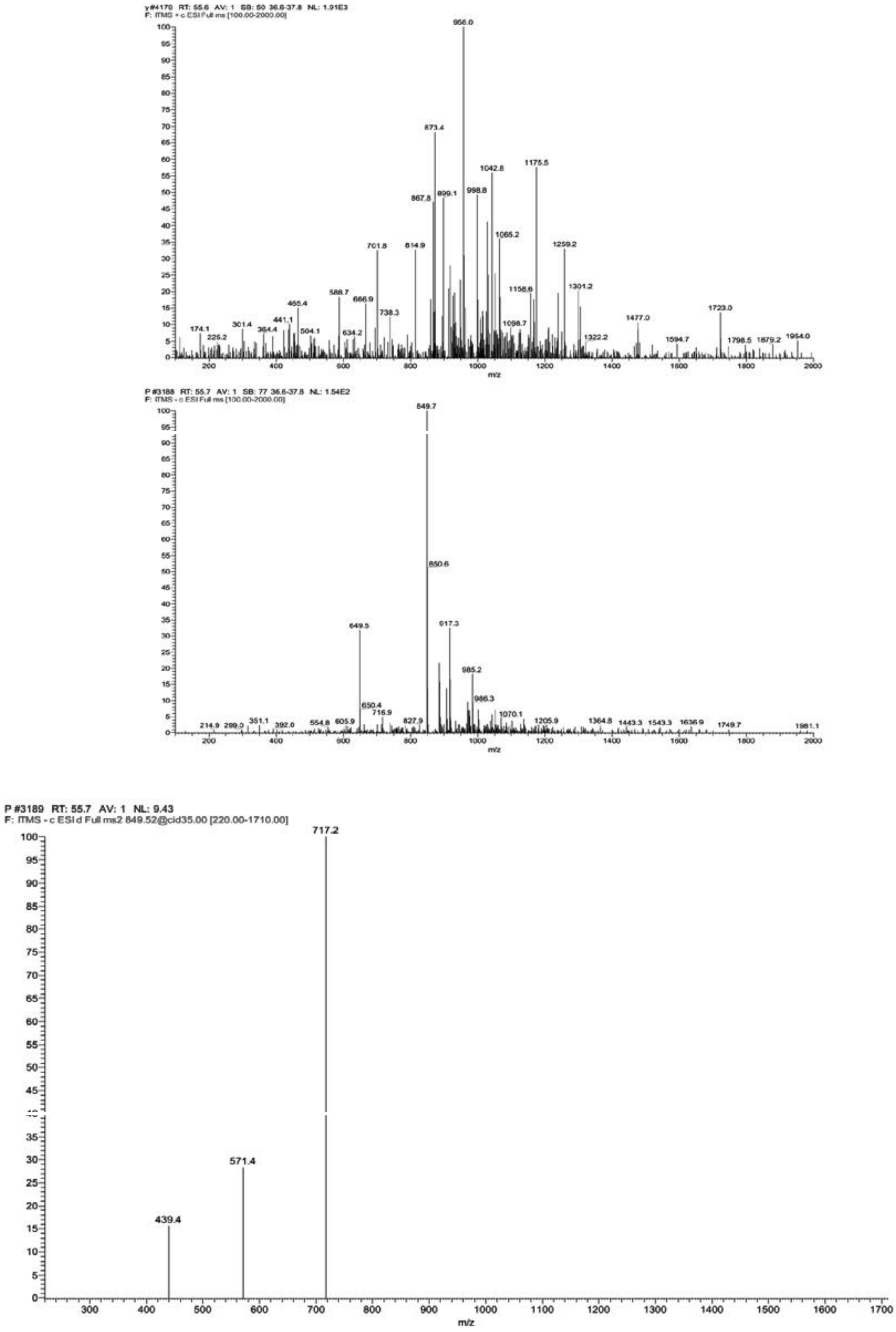

Figure 19: MS spectra of compound **19**

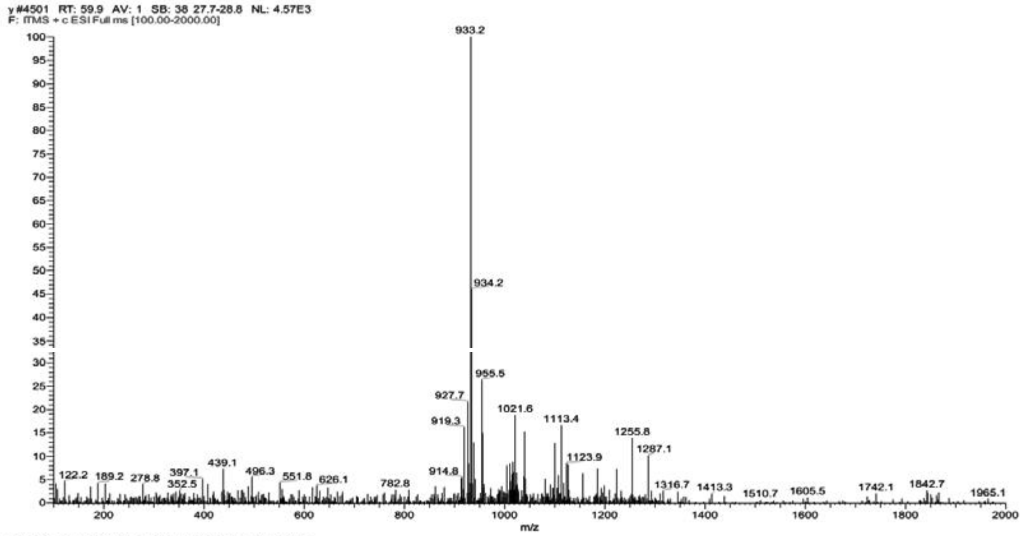
y #4501 RT: 59.9 AV: 1 SB: 38 27.7-28.8 NL: 4.57E3
F: ITMS + c ESI Full ms [100.00-2000.00]
933.2
934.2
955.5
927.7
919.3
1021.6
1113.4
1255.8
1287.1
1123.9
914.8
122.2
189.2
278.8
397.1
352.5
439.1
496.3
551.8
626.1
782.8
1316.7
1413.3
1510.7
1605.5
1742.1
1842.7
1965.1
m/z

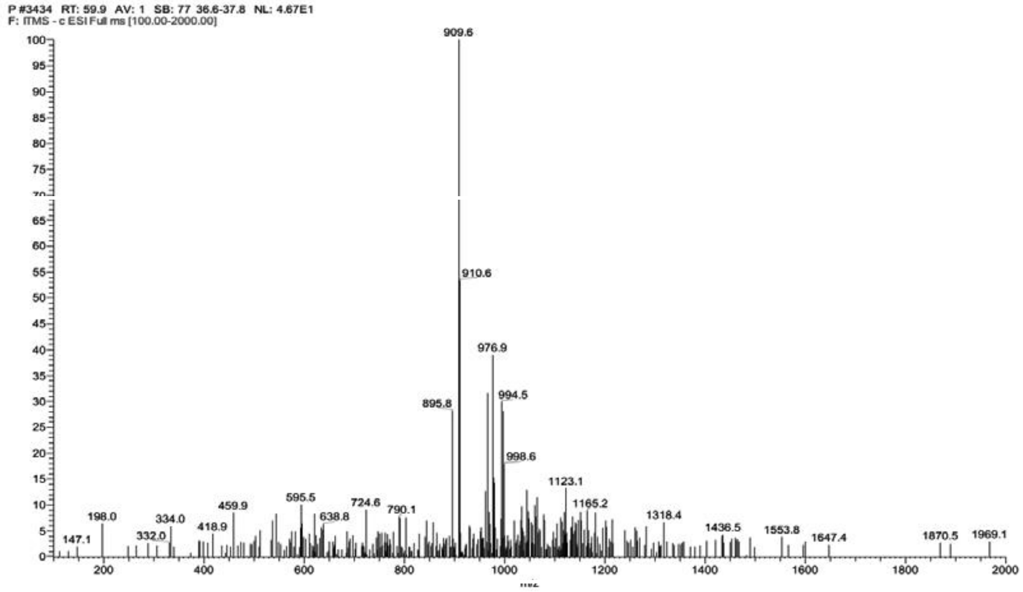
P #3434 RT: 59.9 AV: 1 SB: 77 36.6-37.8 NL: 4.67E1
F: ITMS - c ESI Full ms [100.00-2000.00]
909.6
910.6
976.9
994.5
895.8
998.6
1123.1
1165.2
1318.4
1436.5
1553.8
1647.4
1870.5
1969.1
147.1
198.0
332.0
334.0
418.9
459.9
595.5
638.8
724.6
790.1

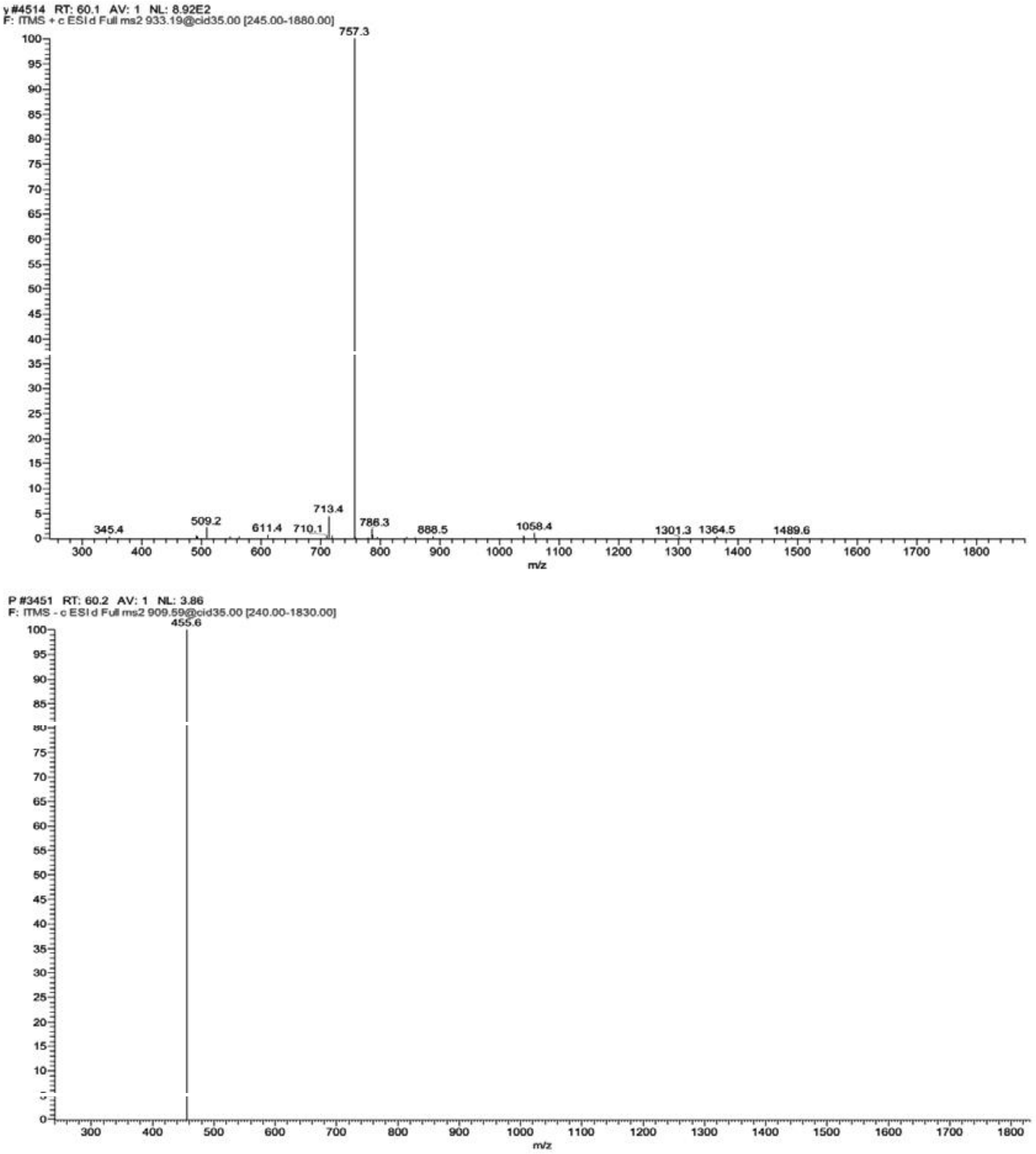

Figure 20: MS spectra of compound **20**

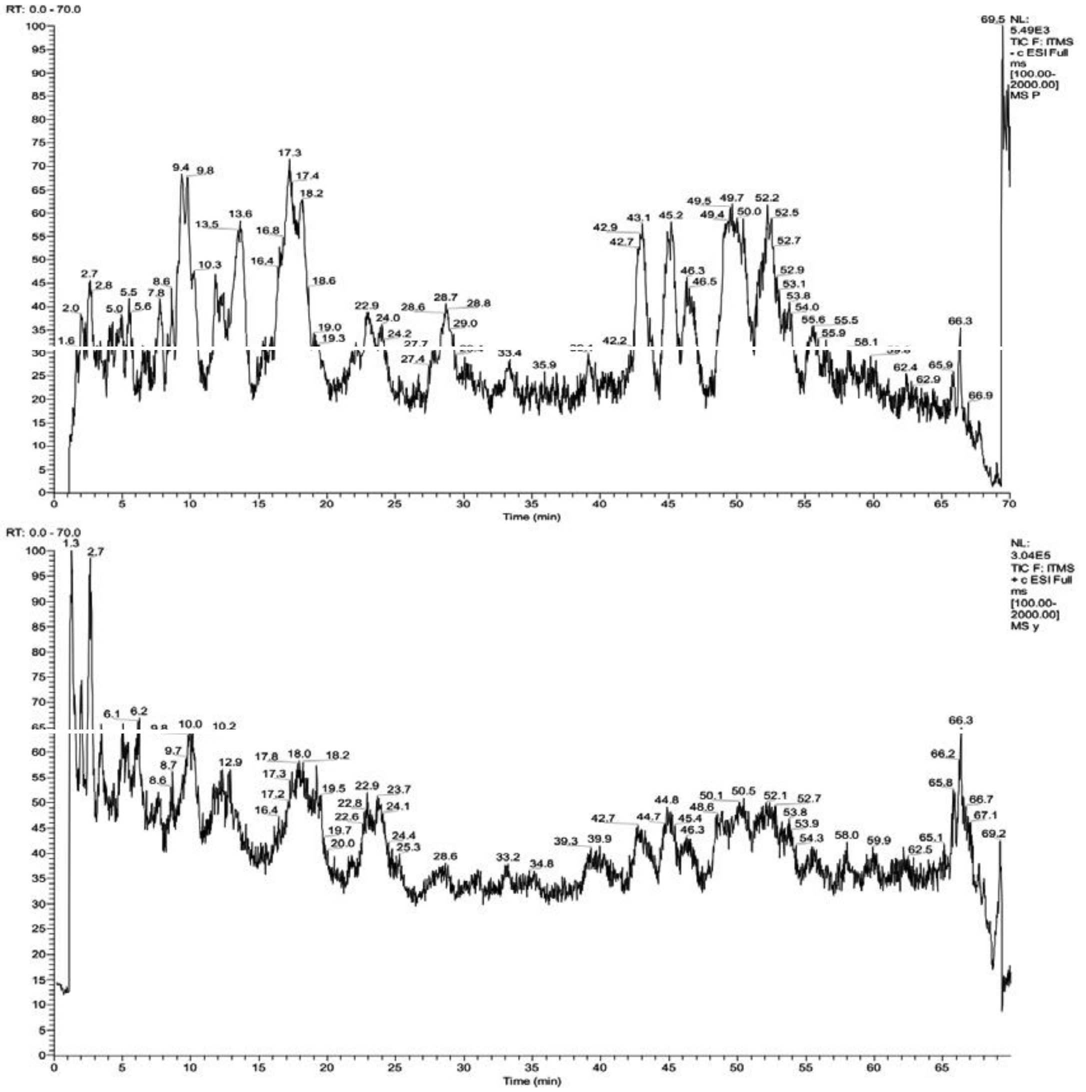

Figure 21: MS total ion current (TIC) chromatogram of TSS

Table 1: Triterpenoid saponins identified in the TSS by HPLC-ESI-MS/MS

Peak No.	t_R (min)	Molecular formula	ESI-MS	ESI-MS^2	Identification
1	7.7	$C_{58}H_{90}O_{28}$	1257 $[M + Na]^+$	1081 $[M + Na-176]^+$<br>935 $[M + Na-176-146]^+$<br>611 $[(M + Na-176)-470]^+$<br>493 $[470+Na]^+$	Stauntoside K
2	9.2	$C_{63}H_{100}O_{30}$	1359 $[M + Na]^+$	889 $[M + Na-470]^+$<br>845 $[(M + Na-470)-44]^+$<br>757 $[(M + Na-470)-132]^+$<br>611 $[(M + Na-470)-132-146]^+$<br>493 $[470+Na]^+$	Yemuoside YM_{28}
3	9.8	$C_{58}H_{92}O_{26}$	1249 $[M+HCOO]^-$<br>1227 $[M + Na]^+$	1081 $[M + Na-146]^+$<br>919 $[M + Na-146-162]^+$<br>757 $[M + Na-470]^+$<br>713 $[(M + Na-470)-44]^+$<br>611 $[(M + Na-470)-132-146]^+$<br>493 $[470+Na]^+$	Glycoside L-H3
4	10.2	$C_{52}H_{82}O_{22}$	1103 $[M+HCOO]^-$<br>1081 $[M + Na]^+$	935 $[M + Na-146]^+$<br>611 $[M + Na-470]^+$<br>493 $[470+Na]^+$	Glycoside L-G1
5	12.3	$C_{52}H_{82}O_{22}$	1103 $[M+HCOO]^-$	919 $[M + Na-162]^+$<br>757 $[M + Na-162-162]^+$<br>713 $[M+Na-162-162-44]^+$	new

			1081 $[M + Na]^+$	347 $[324+Na]^+$	
6	13.6	$C_{59}H_{94}O_{28}$	1249 $[M - H]^-$<br>1273 $[M + Na]^+$	1097 $[M + Na-176]^+$<br>951 $[M + Na-176-146]^+$<br>819 $[M + Na-176-146-132]^+$<br>627 $[(M + Na-176)-470]^+$<br>493 $[470+Na]^+$<br>779 $[M-H-470]^-$	Stauntoside L
7	16.3	$C_{64}H_{104}O_{30}$	1397 $[M+HCOO]^-$<br>1375 $[M + Na]^+$	905 $[M + Na-470]^+$<br>861 $[(M + Na-470)-44]^+$<br>729 $[(M + Na-470-44)-132]^+$<br>627 $[(M + Na-470)-132-146]^+$<br>583 $[(M + Na-470-44)-132-146]^+$<br>493 $[470+Na]^+$	Yemuoside YM_{32}
8	17.9	$C_{59}H_{96}O_{26}$	1265 $[M+HCOO]^-$<br>1243 $[M + Na]^+$	1097 $[M + Na-146]^+$<br>773 $[M + Na-470]^+$<br>729 $[(M + Na-470)-44]^+$<br>493 $[470+Na]^+$	Hederasaponin C
9	19.3	$C_{53}H_{86}O_{22}$	1119 $[M+HCOO]^-$<br>1097 $[M + Na]^+$	951 $[M + Na-146]^+$<br>627 $[M + Na-470]^+$<br>493 $[470+Na]^+$	Hederasaponin D
10	22.7	$C_{58}H_{94}O_{26}$	1251 $[M+HCOO]^-$<br>1229 $[M + Na]^+$	905 $[M + Na-324]^+$<br>861 $[(M + Na-324)-44]^+$<br>787 $[(M + Na-132-146-132-18]^+$<br>347 $[324 + Na]^+$	Yemuoside YM_{35}

11	23.9	$C_{53}H_{86}O_{22}$	1119 $[M+HCOO]^-$<br>1097 $[M + Na]^+$	773 $[M + Na-470]^+$<br>729 $[(M + Na-470)-44]^+$<br>347 $[324 + Na]^+$	Dipsacoside B
12	27.4	$C_{63}H_{100}O_{29}$	1365<br>$[M+HCOO]^-$<br>1343 $[M + Na]^+$	1065 $[M + Na-146-132]^+$<br>873 $[M + Na-470]^+$<br>769 $[(M + Na-132-146-132-18)-146]^+$<br>595 $[M + Na-470-132-146]^+$<br>493 $[470+Na]^+$<br>1039 $[M + HCOO-146-132-18]^-$	Yemuoside YM_{21}
13	31.1	$C_{58}H_{92}O_{25}$	1233 $[M+HCOO]^-$<br>1211 $[M + Na]^+$	1065 $[M + Na-146]^+$<br>933 $[(M + Na-146-132]^+$<br>597 $[M + Na-146-132-146-162-28]^+$<br>475 [470+Na-18]<br>347 [146+162+16+Na]	Yemuoside YM_{10}
14	33.2	$C_{57}H_{90}O_{25}$	1219 $[M+HCOO]^-$<br>1197 $[M + Na]^+$	849 $[M-H-324]^-$<br>609	Yemuoside YM_{24}.

15	45.0	$C_{45}H_{70}O_{16}$	911 $[M+HCOO]^-$<br>889 $[M + Na]^+$	611 $[M + Na-146-132]^+$<br>567 $[M + Na-146-132-44]^+$<br>587 $[M \pm H-146-132]^-$<br>455 $[M \pm H-146-132-132]^-$	Yemuoside YM37
16	48.9	$C_{51}H_{82}O_{20}$	1059 $[M+HCOO]^-$<br>1037 $[M + Na]^+$	585 $[M \pm H-146-132-132-18]^-$	new
17	50.4	$C_{47}H_{74}O_{18}$	925 $[M-H]^-$<br>949 $[M + Na]^+$	773 $[M + Na-176]^+$<br>749 $[M-H-176]^-$<br>603 $[M-H-176-146]^-$<br>471 $[M-H-176-146-132]^-$	new
18	53.7	$C_{41}H_{66}O_{12}$	795 $[M+HCOO]^-$<br>751 $[M + H]^+$	749 $[M-H]^-$	3-O-α-L-rhamnopyranosyl-(1→2)-α-L-arabinopyranosyl-hederagenin
19	55.7	$C_{45}H_{70}O_{15}$	849 $[M-H]^-$<br>873 $[M + Na]^+$	717 $[M-H-132]^-$<br>571$[M-H-132-146]^-$<br>439$[M-H-132-146-132]^-$	3-O-α-L-arabinopyranosyl-(1→3)-α-L-rhamnopyranosyl-(1→2)-α-L-arabinopyranosyl-Akebonic acid
20	60.0	$C_{47}H_{74}O_{17}$	909 $[M - H]^-$<br>933 $[M + Na]^+$	757 $[M + Na-176]^+$<br>611 $[M+ Na-176-146]^+$<br>455 $[M-H-176-146-132]^-$	3β-[(*O*-β-D-glucuronopyranosyl-(1→3)-*O*-[α-L-rhamnopyranosyl-(1→2)]-α-L-arabinopyranosyl)oxy]olean-12-en-28-oic acid
